# Revealing Pathway Complexity and Helical Inversion in Supramolecular Assemblies Through Solvent‐Induced Radical Disparities

**DOI:** 10.1002/advs.202308371

**Published:** 2024-02-04

**Authors:** Haotian Ma, Xiaoxiao Cheng, Gong Zhang, Tengfei Miao, Zixiang He, Wei Zhang

**Affiliations:** ^1^ State and Local Joint Engineering Laboratory for Novel Functional Polymeric Materials Jiangsu Engineering Laboratory of Novel Functional Polymeric Materials Suzhou Key Laboratory of Macromolecular Design and Precision Synthesis College of Chemistry Chemical Engineering and Materials Science Soochow University Suzhou 215123 China; ^2^ Jiangsu Key Laboratory for Chemistry of Low‐Dimensional Materials School of Chemistry and Chemical Engineering Huaiyin Normal University Huaian 223300 China; ^3^ School of Chemical and Environmental Engineering Anhui Polytechnic University Wuhu 241000 China

**Keywords:** helical inversion, pathway complexity, radicals, solvent–solute interaction, supramolecular assembly

## Abstract

New insights are raised to interpret pathway complexity in the supramolecular assembly of chiral triarylamine tris‐amide (TATA) monomer. In cosolvent systems, the monomer undergoes entirely different assembly processes depending on the chemical feature of the two solvents. Specifically, 1,2‐dichloroethane (DCE) and methylcyclohexane (MCH) cosolvent trigger the cooperative growth of monomers with *M* helical arrangement, and hierarchical thin nanobelts are further formed. But in DCE and hexane (HE) combination, a different pathway occurs where monomers go through isodesmic growth to generate twisted nanofibers with *P* helical arrangement. Moreover, the two distinct assemblies exhibit opposite excited‐state chirality. The driving force for both assemblies is the formation of intermolecular hydrogen bonds between amide moieties. However, the mechanistic investigation indicates that radical and neutral triarylamine species go through distinct assembly phases by changing solvent structures. The neutralization of radicals in MCH plays a critical role in pathway complexity, which significantly impacts the overall supramolecular assembly process, giving rise to inversed supramolecular helicity and distinct morphologies. This differentiation in pathways affected by radicals provides a new approach to manipulate chiral supramolecular assembly process by facile solvent–solute interactions.

## Graphical abstract

Pathway complexity and helical inversion of chiral triarylamine assembly is investigated. In different solvents, monomer undergoes isodesmic and cooperative growth separately, and 1D supramolecular assemblies with opposite helicity are constructed. Mechanistic study reveals that compared with radical form in linear alkanes, monomers are neutralized by cycloalkanes, thus triggering a distinct pathway.

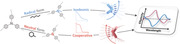

## 1 Introduction

Over the past decades, supramolecular polymerization has been proved to be a fascinating strategy to construct functional materials^[^ ^1^, ^2^, ^3^ ^]^ with miscellaneous properties relying on the interior dynamic nature,^[^ ^4^, ^5^ ^]^ which instead bring challenges in designing and manipulating for application.^[^ ^6^, ^7^ ^]^ Despite extensive utilization in self‐healing materials,^[^ ^8^ ^]^ electroconductive fibers,^[^ ^9^, ^10^ ^]^ photodetectors^[^ ^11^ ^]^ and biomedical materials,^[^ ^12^ ^]^ supramolecular polymers (SPs) yet embody abundant potentials, where the diverse pathways play a key role.^[^ ^13^ ^]^ By virtue of the dynamic behaviors of supramolecular interactions, various inducing sources such as thermo‐treatment,^[^ ^14^ ^]^ kinetic trapping,^[^ ^15^ ^]^ solvation,^[^ ^16^ ^]^ circularly polarized light irradiation^[^ ^17^ ^]^ and stirring^[^ ^18^ ^]^ were capable of triggering different pathways characterized as thermodynamic and kinetic states.^[^ ^19^ ^]^ The stable thermodynamic state and unstable kinetic state usually possess distinct internal structures and display entirely different morphologies.^[^ ^20^ ^]^ Furthermore, the off‐pathway kinetic trapped state is an excellent dormant for living supramolecular polymerization^[^ ^21^ ^]^ to control monomer concentration and obtain SPs of uniform length with active terminals. Thus, a comprehensive interpretation of pathway complexity ought to be accomplished and is vital for further design and regulation of desired SPs or assemblies.

Particularly, SPs of helical arrangement or endowed with chiral information exhibit various specific characteristics and therefore draw lots of attention these years.^[^ ^22^, ^23^, ^24^, ^25^ ^]^ With chirality introduced, various intriguing properties would appear such as chiral amplification^[^ ^26^ ^]^ (“Sergeants and Soldiers principle”^[^ ^27^, ^28^, ^29^ ^]^ and Majority rules^[^ ^30^, ^31^ ^]^), symmetry breaking,^[^ ^32^ ^]^ chirality‐induced spin‐selectivity (CISS) effect,^[^ ^33^ ^]^ chain capper and sequestrator effect,^[^ ^34^ ^]^ etc. These characteristics motivate the investigation of relations between chiral monomer and supramolecular helical structures, chirality transfer in different scales, and even the origination of chirality. Pathways in supramolecular polymerization with chiral manifestation have been scrutinized using functional monomers to manipulate the chiral structures in different scales.^[^ ^35^, ^36^, ^37^ ^]^ As a typical disc‐shape monomer in supramolecular polymerization, triarylamine (TAA) derivatives are widely investigated as hole transporting materials.^[^ ^38^, ^39^ ^]^ The supramolecular polymerization of TAA derivatives was first discovered by Giuseppone,^[^ ^9^ ^]^ 1D supramolecular polymerization of substituted monoamide and trisamide could be triggered by visible light irradiation in a chlorinated solvent.

The formed fibers were electroactive and were able to undergo a light‐induced healing process. Then, Kim introduced chiral information into achiral TAA monomers utilizing circularly polarized light and ultraviolet light at the gel state.^[^ ^17^ ^]^ The chirality of the resulting assembly was determined by the handedness of circularly polarized light. Inspired by Kim's work, the flexible helical structures of TAA derivatives allow us to access different pathways with chiral manifestation.

**Scheme 1:**
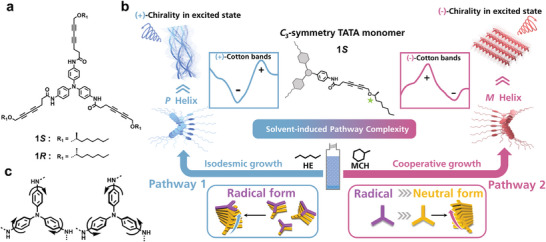
a) Chemical structures of monomer 1*S* and 1*R*. b) Schematic representation of the two pathways in different solvents and the corresponding supramolecular polymerization processes. c) Illustration of two opposite rotating directions of central phenyl moieties.

Herein, we reported the exploration of pathway complexity in supramolecular assembly of chiral disc‐like TAA monomer 1*S* (**Scheme** **1**a,b). We strategically position stereocenters away from the center to balance the effect of chirality transference and intrinsic helical stacking of the center, so that more flexible helical structures are anticipated in solution. The structure is nearly planar so it can easily stack along the axis vertical to the plane through *π*–*π* interactions. The supramolecular chirality originates from the proximity of phenyl moieties, the clockwise or counterclockwise arrangement of three phenyl rings in a single monomer determines the helical direction of assembly as left‐handed or right‐handed orientations (Scheme 1c). The formed assemblies exhibit opposite CD signals in cosolvent (a combination of good and poor solvent) where the poor solvent is changed from MCH to HE. The inversed helical arrangement of monomers within the assembly were scrutinized. Thermodynamic measurements disclose two pathways in cycloalkane and linear alkane governed by cooperative and isodesmic mechanisms, respectively. The distinct hierarchical self‐assembly morphology of two aggregates were revealed utilizing transmission electron microscope (TEM) and atomic force microscope (AFM). Fourier transform infrared spectroscopy (FT‐IR) and concentration‐dependent nuclear magnetic resonance (NMR) measurements demonstrate that hydrogen bonds between amide and carbonyl groups facilitate 1D supramolecular polymerization of 1*S*. Further theoretical calculations were performed within a trimer structure using Molecular Orbital Package^[^ ^40^ ^]^ (MOPAC) and Orca.^[^ ^41^, ^42^, ^43^, ^44^, ^45^ ^]^ More investigation in spectra suggested that radical species were critical in two pathways. Then, we clarified the principles of two entirely distinct pathways where the radical neutralization condition determined the whole process. Extra control experiments also corroborate our assertions. Additionally, opposite circular polarized luminescence (CPL) signals of two aggregates in solution indicate distinct asymmetric environments at excited states corresponding to different helical structures.

## 2 Results and Discussion

### 2.1 Kinetic and Thermodynamic Analysis of Pathways

TATA monomer 1*S* was prepared by a series of reactions similar to Kim's research. The chiral terminal groups were introduced through a Williamson ether synthesis method. Then an asymmetric 1,3‐diyne coupling reaction was applied to generate diacetylene moieties. ^1^H NMR and mass spectroscopy (MS) measurements confirmed the successful synthesis of the chiral monomer 1*S* (Figures S1 and S2, Supporting Information). Thermal analysis revealed the existence of order‐to‐disorder transition around room temperature (Figure S3, Supporting Information). Then, the self‐assembly properties of 1*S* monomer were investigated thoroughly in solution. It turns out that monomer 1*S* dissolves in most organic solvents except for linear alkanes and cycloalkanes with significantly low absorbance in UV–vis spectra (Figure S4, Supporting Information). However, the solubility of 1*S* in alkanes was so poor that precipitation occurred quickly instead of self‐assembly. This phenomenon probably ascribes to the huge divergence between solvation of core and terminals in alkane solvents.

**Figure 1:**
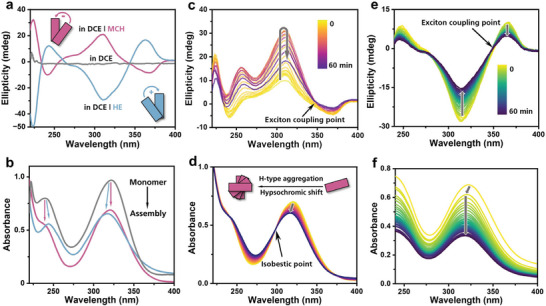
a) CD and b) UV spectra of 1*S* self‐assembly in DCE/MCH and DCE/HE solutions (3% volume fraction of DCE) compared with monomer state in single DCE. c) Time‐dependent CD and UV d) spectra of 1*S* self‐assembly in DCE/MCH. The arrows indicate the time‐dependent changes. e) Time‐dependent CD and UV f) spectra of 1*S* self‐assembly in DCE/HE. The arrows indicate the time‐dependent changes. All monomer concentrations were 32 µm.

Therefore, “good and poor solvent” strategy was applied to trigger supramolecular assembly of 1*S*. Unexpectedly, 3% DCE is sufficient to provoke polymerization both in MCH and HE. As shown in **Figure** **1**a,b, significant reduction of UV absorbance in binary solvents compared with that in good solvent indicates the formation of assemblies. The UV absorbance peak at 320 nm, attributing to triarylamine moiety, goes through a hypsochromic shift, which represents H‐type aggregation of chromophores. The corresponding CD splitting bands originate from the exciton coupling of triarylamine chromophores implying 1D discotic stacking with certain handedness. The left‐ or right‐handed order of monomers within the assembly generate entirely opposite cotton bands in CD spectra, indicating the emergence of supramolecular helicity which is independent of configuration chirality of monomer. The molecule chirality in terminal alkoxyl groups is successfully transferred through diacetylene moieties to the triarylamine centers. It turns out that the poor solvent plays a crucial role in the supramolecular assembly process and is capable of directing different pathways by controlling the inner arrangement of monomers. Furthermore, the enantiomer 1*R* was synthesized and investigated with the same approach. The CD curves were found to be opposite to those of 1*S* in the same solvent (Figure S5, Supporting Information). Therefore, the following research was mainly performed with 1*S* monomer unless otherwise specified. In addition, traces of water may have an impact on the internal structure of supramolecular assemblies.^[^ ^46^ ^]^ Therefore, we tested the CD spectra of assemblies under different water contents and ruled out this potential influencing factor based on the spectra that showed no changes in the shapes of cotton bands (Figure S6, Supporting Information).

Obviously, the supramolecular assembly of 1*S* in binary solvents environment is endowed with certain kinetic properties due to the solvent‐solute interactions. Figure 1c,d illustrates the time‐dependent revolution of CD and UV signals in DCE/MCH solution. The constant reduction and hypsochromic shift of the maximum absorbance peak in the DCEMCH solution indicate continuous H‐type aggregation. The isobestic point at 298 nm provides additional evidence of supramolecular polymerization process of 1*S* where monomers are gradually attached to the polymers in a uniform fashion. Moreover, the initial increase of CD intensities corroborates homochiral polymerizing stage occurring at the active terminals, and the subsequent decrease implies further self‐assembly process commencing at macromolecular scale which will be discussed later. While in the case of DCE/HE system, reduction of CD intensities (Figure 1e) indicates the macromolecular self‐assembly procedure. However, the lack of CD rising stage at beginning reveals distinct supramolecular assembly processes in cyclo‐ and linear alkane solvents. Strong insolubilizing force of linear alkane results in the absence of isobestic point in time‐dependent UV spectra (Figure 1f). The continuous decrease of both CD and UV intensities probably ascribe to sedimentation of giant aggregate due to the extremely low insolubility in hexane.

**Figure 2:**
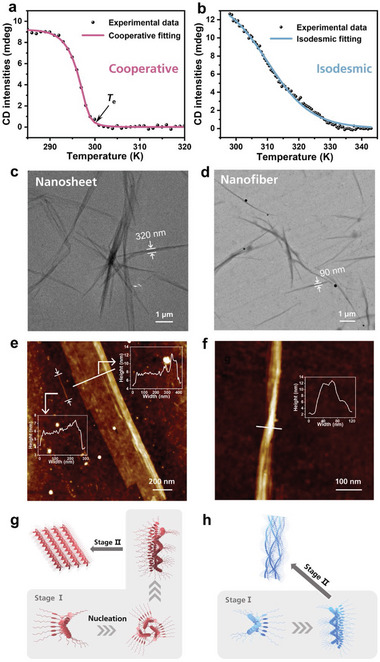
a) CD variations at single point versus temperature during heating process (heating rate: 1 K min^−1^) of 1*S* assembly in DCE/MCH and the corresponding cooperative fitting. *c* = 16 µm. b) CD variations at single point versus temperature during heating process (heating rate: 1 K min^−1^) of 1*S* assembly in DCE/HE and the corresponding isodesmic fitting. *c* = 16 µm. c) TEM and e) AFM images of aggregate in DCE/MCH. d) TEM and f) AFM images of aggregate in DCE/HE. g) Schematic illustration of formation of hierarchical nanostructures of aggregate in DCE/MCH. h) Schematic illustration of formation of hierarchical nanostructures of aggregate in DCE/HE.

Next, the mechanisms of formation of supramolecular assemblies were investigated carefully. In DCE/MCH, a slow cooling process yields distinct single bands in CD spectra (Figure S7a, Supporting Information) which differed from the bisignate cotton bands observed during kinetic assembly at room temperature. The derived curve of degree of aggregation based on CD intensities (Figure S7c, Supporting Information) demonstrates cooperative growth at higher temperatures and gradually attenuates as the temperature decreases further. This observation, along with scattered UV data points obtained at lower temperatures (Figure S7d, Supporting Information), led to the conclusion that precipitation occurred in this system after slow cooling. While in DCE/HE, we were unable to achieve CD active assemblies through slow cooling process (Figure S7b, Supporting Information). Therefore, it can be concluded that thermodynamic assembly cannot be achieved via slow cooling in this system. Consequently, the dissociation process of kinetic assembly was monitored during a slowly heating procedure (**Figure** **2**a,b). The dissociation of 1*S* assemblies occurs as the CD intensities decrease in a nonlinear fashion both in MCH and HE (Figures S8 and S9, Supporting Information). Applying data to a mass balance model^[^ ^47^ ^]^ reveals cooperative and isodesmic fitting separately. The result indicates that poor solvents not only influence the helical direction within the kinetic assemblies, but also have an impact on the mechanism of supramolecular polymerization.

### 2.2 Morphology and Molecular Packing Analysis

To obtain a better comprehension of the pathway complexity, the nanostructures of two assemblies were characterized using TEM and AFM. Unfortunately, explicit chiral structures such as helical fibers, twisted ribbons, or tangled strands were not discovered in this case. However, two aggregates assembled in DCE/MCH and DCE/HE exhibit distinctive morphology as depicted in Figure 2c–f and Figures S10–S13 (Supporting Information). In DCE/MCH solution, slender nanobelts with uniform length were spotted in TEM images. AFM measurement further reveal that they are composed of straight nanowires aligning compactly in the lateral direction, which indicates a hierarchical assembling process consistent with the time‐dependent CD spectra discussed above. Monomers initially undergo 1D supramolecular polymerization to afford single nanowires and then nanowires are combined laterally to fabricate thin nanobelts (Figure 2g). A similar hierarchical assembly process was discovered in DCE/HE solution. SEM and AFM images disclose short spike‐like nanostructures composed of multiple nanofibers as is shown in the up‐scaling image. These soft nanofibers intertwine randomly without obvious chiral direction (Figures 2h), which is different from ordered arrangement of rigid nanowires in DCE/MCH, implying unequal forces applied by different poor solvents in hierarchical assembling stages.

**Figure 3:**
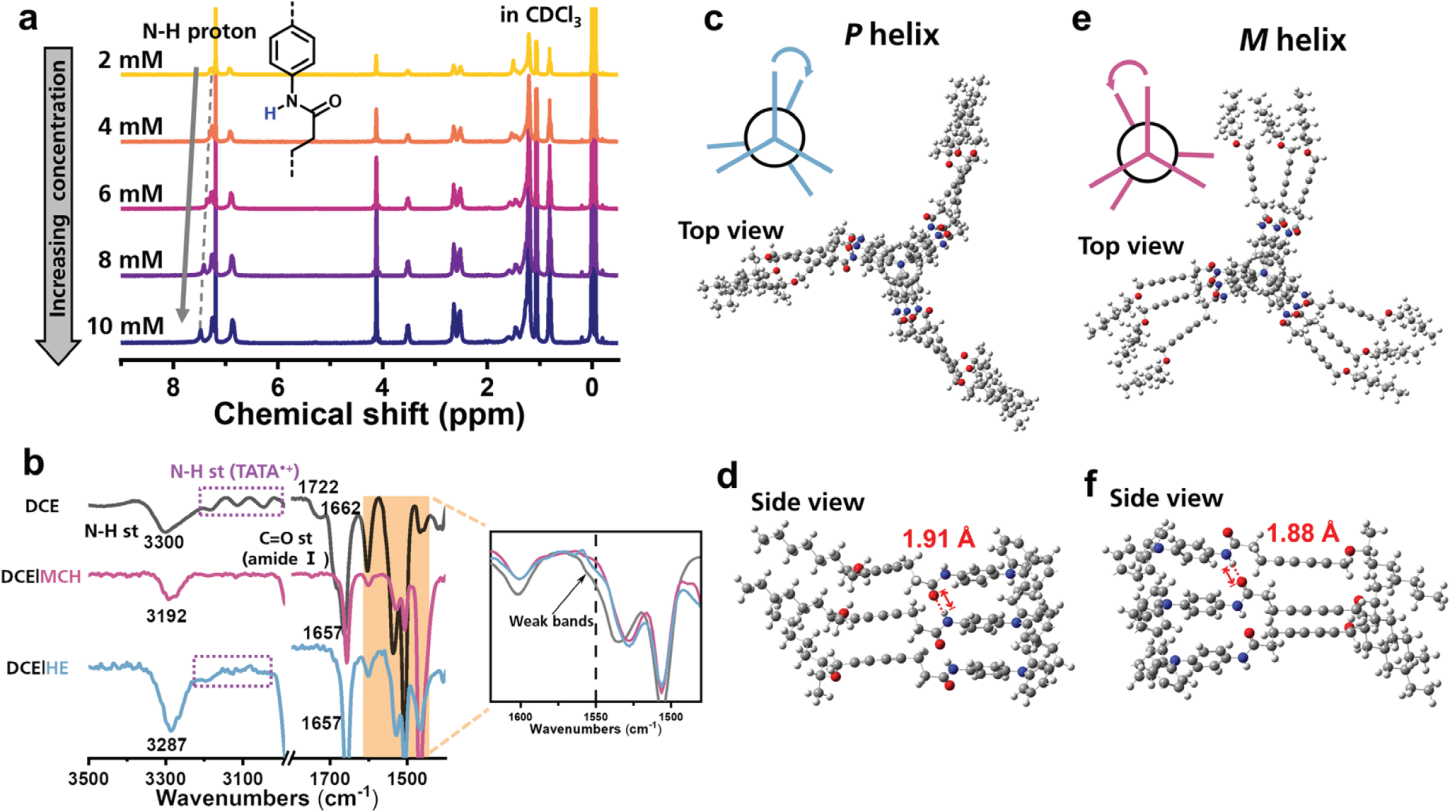
a) Concentration‐dependent NMR signals of 1*S* in deuterated chloroform, arrow indicates the shift of amide protons. b) FT‐IR spectra of 1*S* in DCE, DCE/HE and DCE/MCH with 3% volume fraction. *c* = 32 µm. The magnified image shows the minor differences of weak bands ≈1550 cm^−1^. c) Top and d) side views of optimized structures of 1*S* trimers with *P* helix directions. e) Top and f) side views of optimized structures of 1*S* trimers with *M* helix directions.

Subsequently, interactions between monomers were then investigated through a series of experiments. Concentration‐dependent NMR measurements in deuterated chloroform (**Figure** **3**a) confirm the intermolecular hydrogen bonds. The amide proton peak at 7.2 ppm shifts to lower field with concentration increases from 2 to 10 mm. FT‐IR spectra also confirm the formation of hydrogen bonds as is shown in Figure 3b, the N–H vibration peak locate ≈3300 cm^−1^ and carbonyl peaks appear at 1657 cm^−1^. Moreover, dissociation of hydrogen bonds by adding trifluoroacetic acid (TFA) results in completely loss of chirality (Figures S14 and S15, Supporting Information), which demonstrates that the supramolecular helicity of aggregate relies on intermolecular hydrogen bonds formation between amide and carbonyl groups.

Moreover, theoretical calculations were performed utilizing MOPAC^[^ ^40^ ^]^ to analyze a trimer structure with *P* or *M* helical arrangement, and the corresponding CD spectra were predicted using Orca.^[^ ^41^, ^42^, ^43^, ^44^, ^45^ ^]^ The computational results were analyzed by taking advantage of the Multiwfn program.^[^ ^48^ ^]^ Figure 3c–f shows the optimized structures of 1*S* trimer, and the related CD spectra were calculated and depicted in Figure S16 (Supporting Information). The *P* helical trimer shows right‐handed helical arrangement of monomers and diacetylene moieties tend to form cross‐conformations to each other which reduces the whole trimer size. The calculated CD spectrum shows positive and negative cotton bands which are in line with our experimental results in DCE/HE and DCE/MCH. The noticeable distance between carbonyl oxygen and amino hydrogen which is ≈1.9 Å claims the formation of hydrogen bonds.

### 2.3 Radical Investigation and Insights into Principles of Distinct Supramolecular Assembly Process

**Figure 4:**
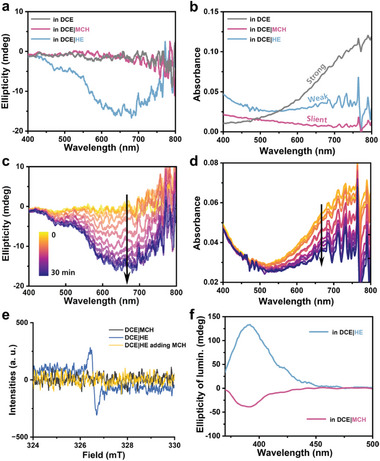
a) CD and b) UV spectra in long‐wavelength region of 1*S* assemblies in different solvents compared with monomer state in single DCE. c) Time‐dependent CD and d) UV spectra in long‐wavelength region of 1*S* assembly in DCE/HE. e) EPR spectra of assembly in DCE/HE and DCE/MCH (*c* = 1 mm), and the quenching sample by adding MCH into the DCE/MCH assembly. f) CPL spectra of 1*S* assemblies in DCE/MCH and DCE/HE. All monomer concentrations were 32 µm, 3% volume fraction of DCE unless otherwise declared.

Finally, more profound investigations ought to be accomplished in order to interpret the principles thoroughly. The FT‐IR spectra not only reveal the formation of hydrogen bonds, but also provide more important information near N–H stretching vibration region where weak absorbance bands are spotted ≈3200 cm^−1^ in DCE and DCE/HE solutions (Figure 3b; Figure S17, Supporting Information). These bands are attributed to the presence of radical species of TATA monomer.^[^ ^49^ ^]^ The weak bands at 1550 cm^−1^ also correspond with the phenomenon that radical species are present in DCE and DCE/HE, but absent in DCE/MCH. To further investigate radical species, CD and UV spectra at long wavelength of 1*S* assembly in three solutions were measured and illustrated in **Figure** **4**a,b. The absorbance peak ≈800 nm indicates the presence of radicals in DCE and DCE/HE. Moreover, the negative bands in CD spectrum further demonstrate that the radical species containing supramolecular helical structures in DCE/HE system, which is consistent with negative CD signal at 311 nm. The increasing CD intensity and decreasing absorbance in time‐dependent CD and UV spectra (Figure 4c,d) reveal the growing homochiral structures of radical species in DCE/HE, implying that the distinct pathways in different poor solvents are interrelated to the formation of radical species. Additionally, the electron paramagnetic resonance (EPR) spectra (Figure 4e) confirm the existence and absence of radicals in DCE/HE and DCE/MCH at high concentrations. The introduction of MCH leads to the disappearance of the radical signal, indicating a significant disparity in the occurrence or absence of radicals between these two solvents.

**Scheme 2:**
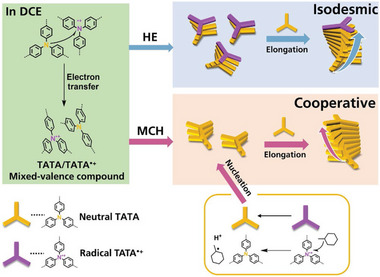
Elucidation of mechanism about pathway complexity in different solvents under the influence of radicals.

According to the above analysis, we then provide our hypothesis in principles of pathway complexity of 1*S* assembly utilizing radical effect (**Scheme** **2**). First the 1*S* monomers partly form radical species in chlorinated solvent such as DCE under natural sunlight. These TATA radicals could be stabilized by binding with neutral TATA molecules in close proximity thus giving rise to mixed‐valence compound^[^ ^50^ ^]^ TATA/TATA^•+^. When HE is added into DCE solution, the supramolecular polymerization process is triggered by strong solvophobic force exerted by poor solvent. The TATA/TATA^•+^ mixed‐valence compound can act as nuclei to initiate isodesmic growth in one‐dimensional fashion. The terminal radicals remain active thus displaying radical signals in FT‐IR as well as CD and UV spectra, which is consistent with increasing CD signals in time‐dependent spectra. But different procedure would occur when MCH is added. The radicals are quenched by the tertiary carbon of MCH, causing the loss of radical signals in FT‐IR, CD and UV spectra. Therefore, the neutral TATA monomers go through nucleation and elongation process in line with the cooperative mechanism since no radical nuclei can serve as the initiator in DCE/MCH.

More importantly, the formation of radicals can affect the helical structures within the assembly. As is shown Figure S18 (Supporting Information), the modified spatial structures of trimers reveal variations in the C─N─C bond angle within the central region of *P* and *M* helical structures. The bond angle measures ≈118° in the *P* structure, while slightly smaller at 117° in the *M* structure. According to this phenomenon, we suggest that when triphenylamine is in its radical state, the nitrogen atom at its center adopts sp^2^ hybridization without experiencing electron repulsion from a lone pair of electrons. Consequently, it exhibits an almost planar structure and tends to stack in a *P* helix conformation. However, in the neutral form of triphenylamine, the nitrogen atom is sp^3^ hybridization with the accompany of a lone pair of electrons. This leads to a bowl‐shaped structure where stack into *M* helix with a small bond angle becomes feasible. In summary, it can be concluded that the presence or absence of radicals can influence the helical orientation of stacking through modulation of C─N─C bond angles at the central region, therefore affecting chiral expressions.

Subsequently, more experiments were performed to authenticate the principles. Figure S19 (Supporting Information) shows the time‐dependent CD spectra of 1*S* assembly in DCE/HE with the addition of MCH. The stationary CD and UV signals indicate the termination of supramolecular polymerization because MCH quench active radicals in DCE/HE solution and suspend the polymerization process. However, excess amount of radicals will hamper the supramolecular polymerization in DCE/MCH as is shown in Figure S20 (Supporting Information). Some radicals are not quenched immediately and go through the same aggregation process as in HE, resulting in considerable decrease of CD intensities. Furthermore, control experiments using different cyclic and linear alkanes were performed (Figure S21, Supporting Information). In branched linear alkanes with tertiary carbons (isooctane and 2‐methylpentane), no obvious chiral assembly was obtained because the radicals were quenched and unable to assemble. Similar to MCH, 1*S* in decalin exhibited cotton bands upon assembly while cyclohexane without tertiary carbons failed to generate cotton bands and displayed a negative signal band instead. This demonstrates that the presence or absence of tertiary carbons plays a crucial role in helical assembly of monomers within linear and cyclic alkanes respectively. Linear alkanes rely on radical‐mediated assembly which cannot occur when tertiary carbons are present while cyclic alkanes require tertiary carbons to quench radicals and facilitate helical assembly of neutral monomers.

Moreover, since triarylamine derivatives are conventional fluorescent materials, the luminescence of 1*S* assembly in solution might interfere the circular dichroism and intensities of absorbance, hence hamper the measurement and analysis of chirality of assemblies. In order to examine this effect, fluorescence‐detected circular dichroism (FDCD) measurements were performed and the CD‐silent spectra (Figures S22 and S23, Supporting Information) verify that fluorescence almost have no impact on absorbance and circular dichroism. Additionally, the self‐assembly in DCE/MCH and DCE/HE exhibits considerable CPL emissions (Figure 4f; Figures S24 and S25, Supporting Information), and the opposite signals are consistent with the simultaneously reversed helicity of assemblies in excited state.

## 3 Conclusion

In this work, we demonstrated pathway complexity of supramolecular assemblies consisting of chiral TATA monomer 1*S* regulated by distinct solvents. The two pathways enable the formation of reversed helical structures by different growing mechanisms. The hierarchical self‐assembly process comprises 1D packing stage driven by *π*–*π* stacking and hydrogen bonding interactions, and further assembling stage to fabricate massive aggregates under isodesmic or cooperative pathways. As a result, the supramolecular helicity is independent of the configuration of the stereocenter of monomer. The radical neutralization ability of MCH is crucial to induce distinct hierarchical supramolecular self‐assembly process compared with HE. Furthermore, this radical‐associated principle of pathway complexity distinguished by alkane solvents was verified by control experiments. Therefore, the binary solvent‐directed mechanism of radicals of triarylamine‐based supramolecular assembly pathways is realized. We believe it will provide a novel approach to predict and direct pathway complexity of triarylamine derivatives, enable precisely control of supramolecular assembly process and pave the way for design and fabrication of chiral functional materials.

## Conflict of Interest

The authors declare no conflict of interest.

## Supporting information

Supporting Information

## Data Availability

The data that support the findings of this study are available in the supplementary material of this article.
