# Supplementary material for: Revealing Pathway Complexity and Helical Inversion in Supramolecular Assemblies Through Solvent‐Induced Radical Disparities
Source: Adv Sci (Weinh). 2024 Feb 4;11(14):2308371. doi: 10.1002/advs.202308371 (PMC11005740; doi:10.1002/advs.202308371)
Supplement: Supplementary file 1 — Supporting Information [file ADVS-11-2308371-s001.pdf]

ADVANCED SCIENCE
Open Access

Supporting Information

for *Adv. Sci.*, DOI 10.1002/advs.202308371

Revealing Pathway Complexity and Helical Inversion in Supramolecular Assemblies Through Solvent-Induced Radical Disparities

Haotian Ma, Xiaoxiao Cheng, Gong Zhang, Tengfei Miao, Zixiang He and Wei Zhang**

Supporting Information

Revealing Pathway Complexity and Helical Inversion in Supramolecular Assemblies through Solvent-induced Radical Disparities

Haotian Ma[a], Xiaoxiao Cheng[a], Gong Zhang*[a], Tengfei Miao[c], Zixiang He[a] and Wei Zhang*[a,b]

[a] H. Ma, Dr. X. Cheng, Dr. G. Zhang, Z. He, Prof. W. Zhang
State and Local Joint Engineering Laboratory for Novel Functional Polymeric Materials, Jiangsu Engineering Laboratory of Novel Functional Polymeric Materials, Suzhou Key Laboratory of Macromolecular Design and Precision Synthesis, College of Chemistry, Chemical Engineering and Materials Science, Soochow University
Suzhou, 215123 (China)
E-mail: gzhang177@suda.edu.cn, weizhang@suda.edu.cn

[b] Prof. W. Zhang
School of Chemical and Environmental Engineering, Anhui Polytechnic University
Wuhu, 241000 (China)

[c] Dr. T. Miao
Jiangsu Key Laboratory for Chemistry of Low-Dimensional Materials, School of Chemistry and Chemical Engineering, Huaiyin Normal University
Huaian, 223300 (China)

Table of Contents

Materials 1
Experimental Procedures 1
Synthesis of (*S*)-2-(prop-2-yn-1-yloxy)octane (1) 1
Synthesis of ethyl pent-4-ynoate (2)............. 1
Synthesis of ethyl (*S*)-8-(octan-2-yloxy)octa-4,6-diynoate (3)... 1
Synthesis of ethyl (*S*)-8-(octan-2-yloxy)octa-4,6-diynoic acid (4) 2
Synthesis of **1*S*** monomer *N*,*N'*,*N''*-(nitrilotris(benzene-4,1-diyl))tris(8-(((*S*)-octan-2-yl)oxy)octa-4,6-diynamide) (5) 2
Characterization and Methods 2
Supplemented Figures 3
Figure S1. Characterization of the **1*S***. 3
Figure S2. Mass spectroscopy measurement of **1*S*** monomer. 3
Figure S3. DSC measured curves of **1*S*** 3
Figure S4. UV-vis spectra of **1*S*** monomer in various single solvents 4
Figure S5. CD and UV spectra of **1*R*** assemblies. 4
Figure S6. The CD spectra of **1*S*** assembly in (a) DCE/MCH and (b) DCE/HE with various water content in poor solvent......................... 4
Figure S7. Temperature-dependent CD spectra of **1*S*** assembly in DCE/MCH and DCE/HE during a slow cooling process with different cooling rates. 5
Figure S8. Dissoication experiments of **1*S*** assemblies in DCE/MCH (3%, v/v) solutions. 5
Figure S9. Dissoication experiments of **1*S*** assemblies in DCE/HE (3%, v/v) solutions 6
Figure S10. TEM images of **1*S*** aggregates assembled in DCE/MCH (3%, v/v) 6
Figure S11. AFM images of **1*S*** aggregates assembled in DCE/MCH (3%, v/v). 6
Figure S12. TEM images of **1*S*** aggregates assembled in DCE/HE (3%, v/v) 7
Figure S13. AFM images of **1*S*** aggregates assembled in DCE/HE (3%, v/v). 7
Figure S14. CD and UV spectra of **1*S*** assembly in DCE/MCH before and after adding TFA to dissociate hydrogen bonds..... 7

Figure S15. CD and UV spectra of **1*S*** assembly in DCE/HE before and after adding TFA to dissociate hydrogen bonds................ 8
Figure S16. Calculated CD spectra of modified trimers with *P* helical and *M* helical arrangement of central phenyl rings.............. 8
Figure S17. IR spectra of **1*S*** assembly in DCE/MCH and DCE/HE with different concentrations. 9
Figure S18. The schematic representation of C-N-C bond angles in P and M helical structures. 9
Figure S19. Time-dependent CD and UV spectra of **1*S*** assembly in DCE/HE after MCH addition. 9
Figure S20. CD and UV spectra of **1*S*** assembly prepared with monomer solution irradiated 10
Figure S21. CD spectra of **1*S*** in DCE and various poor solvents. 10
Figure S22. FDCD spectrum and corresponding luminescence of **1*S*** assemblies in DCE/MCH............................. 11
Figure S23. FDCD spectrum and corresponding luminescence of **1*S*** assemblies in DCE/HE. 11
Figure S24. The g_{lum} values and DC spectrum of **1*S*** assemblies in DCE/MCH 11
Figure S25. The g_{lum} values and DC spectrum of **1*S*** assemblies in DCE/HE....................... 12
Figure S26. TEM images of **1*S*** in single DCE solvent (*c* = 32 μM)...................................... 12
Figure S27. Photos of initial assembly solution right after the addition of poor solvent and the assembly solution after overnight incubation12
References 13
Appendix 14
P helical structure 14
M helical structure 18

Supplemental experimental procedures

Materials

(*R*) and (*S*)-Octan-2-ol (99%, Alfa), sodium hydride (NaH, AR, Enox), 3-bromoprop-1-yne (80% with toluene stable with MgO, Energy), pent-4-ynoic acid (98%, Leyan), sulfuric acid (AR, Enox), copper(I) iodide (CuI, 98%, Aladdin), nickel chloride hexahydrate (99.9%, Aladdin), *N,N,N′,N′*-tetramethylethylenediamine (TMDEA, 99%, Energy), *N,N*-bis(4-aminophenyl) benzene-1,4-diamine (98%, Leyan), 1-hydroxybenzotriazole monohydrate (HOBT, 99%, Admas beta), 1-(3-dimethylaminopropyl)-3-ethylcarbodiimide hydrochloride (EDCI, 98%, Macklin), sodium hydroxide (AR, Enox) and triethylamine (TEA, AR, Enox) were used as received.

Experimental Procedures

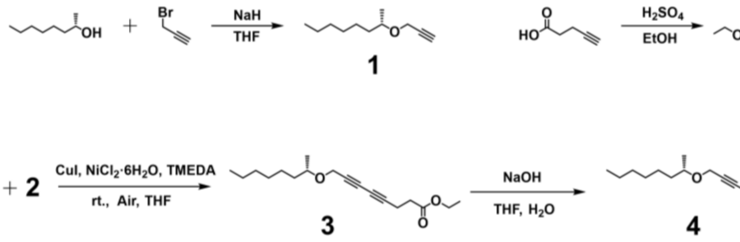

Scheme S1. Synthetic route of monomer **1*S***.

Synthesis of (*S*)-2-(prop-2-yn-1-yloxy)octane (1)
NaH (0.36 g, 15 mmol) was dispersed in 20 mL THF with argon protection and stirred at 0 °C. (*S*)-Octan-2-ol (1.3 g, 10 mmol) was dissolved in THF and added dropwise into the dispersion, then stirred for 30 min. 3-Bromoprop-1-yne (1.43 g, 12 mmol) was then added slowly into the reaction and then warmed to room temperature. After 2 h, the reaction is completed and quenched by adding saturated NH_4Cl slowly. The mixture was extracted with ethyl acetate and dried over anhydrous $MgSO_4$. The crude product was purified by flash column chromatography (petroleum ether/ethyl acetate = 10:1) and concentrated to obtain the product

(light yellow liquid, 62% yield). ^{1}H NMR (300 MHz, DMSO-d_6): δ = 4.90 (hept, *J* = 6.1, 1H), 4.52 (s, 1H), 1.54 (m, 2H), 1.25 (q, *J* = 3.3, 9H), 1.20 (d, *J* = 6.3, 3H), 0.84 (m, 3H).

Synthesis of ethyl pent-4-ynoate (2)

Pent-4-ynoic acid (1 g, 10.2 mmol) was dissolved in 30 mL methanol, then 0.4 mL H_2SO_4 was added. The mixture was stirred for 2 h and then diluted with water, extracted with dichloroethane and concentrated to obtain the product (colorless liquid, 87% yield). ^{1}H NMR (300 MHz, $CDCl_3$) δ = 5.30 (s, 1H), 3.70 (s, 3H), 2.56 (m, 4H).

Synthesis of ethyl (*S*)-8-(octan-2-yloxy)octa-4,6-diynoate (3)

The synthesis of unsymmetrical 1,3-diynes[1]. CuI (9.5 mg, 0.05 mmol) and $NiCl_2 \cdot 6H_2O$ (12 mg, 0.05 mmol) were added into 4 mL THF and stirred for 30 min. Then TMEDA (30 μL, 0.2 mmol) was added. After the mixture turned green, compound **1** (0.17 g, 1 mmol) and **2** (0.56 g, 5 mmol) were added into the reaction and stirred overnight at room temperature. After reaction completed, the solid was filtered off. The liquid was concentrated and purified by flash column chromatography (petroleum ether/ethyl acetate = 8:1) to obtain the product (yellow liquid, 38% yield). ^{1}H NMR (300 MHz, $CDCl_3$) δ = 4.19 (m, 2H), 3.71 (s, 3H), 3.60 (h, *J* = 6.2, 1H), 2.58 (m, 4H), 1.53 (m, 2H), 1.28 (m, 9H), 1.13 (d, *J* = 6.1, 3H), 0.87 (m, 3H).

Synthesis of ethyl (*S*)-8-(octan-2-yloxy)octa-4,6-diynoic acid (4)

Compound **3** (0.3 g, 1 mmol) was dissolved in 20 mL THF/H_2O (1:1, v/v) mixture and NaOH (0.08 g, 2.0 mmol) was added. After stirring at room temperature for 2 h, the pH of mixture was adjusted to 3 by adding HCl. Extracted with ethyl acetate and dried over anhydrous $MgSO_4$ to obtain the product (white solid, 92% yield). ^{1}H NMR (300 MHz, DMSO-d_6) δ = 11.80 (s, 1H), 4.22 (m, 2H), 3.93 (h, *J* = 6.8, 1H), 2.66 (m, 4H), 1.53 (m, 2H), 1.30 (m, 9H), 1.18 (d, *J* = 6.6, 3H), 0.87 (m, 3H).

Synthesis of 1*S* monomer *N*,*N'*,*N''*-(nitrilotris(benzene-4,1-diyl))tris(8-(((*S*)-octan-2-yl)oxy)octa-4,6-diynamide) (5)

N,N-Bis(4-aminophenyl)benzene-1,4-diamine (0.1 g, 0.34 mmol), compound **4** (0.3 g, 1.14 mmol) and 1-hydroxybenzotriazole monohydrate (0.25 g, 1.63 mmol) were dissolved in 10 mL DMF protected by argon, stirred at 0 °C for 30 min. Then, 1-(3-dimethylaminopropyl)-3-ethylcarbodiimide hydrochloride (0.31 g, 1.62 mmol) and triethylamine (225 μL, 1.62 mmol) were added, then the reaction was warmed up to room temperature. After 12 h reaction, the mixture was diluted with water and extracted with ethyl ether. The organic layer was washed with water, dried over anhydrous $MgSO_4$. The crude product was gained by flash column chromatography (DCM/MeOH = 10:1) and then purified by recrystallization in ethanol to obtain the pure product (dark red solid, 24% yield). ^{1}H NMR (300 MHz, DMSO-d_6) δ = 10.00 (s, 1H), 7.54 (d, *J* = 8.5, 2H), 6.97 (d, *J* = 8.9, 2H), 4.29 (m, 2H), 3.56 (m, 1H), 2.68 (d, *J* = 6.5, 2H), 1.45 (d, *J* = 20.0, 1H), 1.30 (s, 8H), 1.11 (d, *J* = 6.1, 4H), 0.90 (m, 3H).

The **1*R*** monomer was synthesized through the same procedures by changing the materials to the corresponding enantiomers.

Characterization and Methods

^{1}H NMR spectra were recorded on a nuclear magnetic resonance instrument (300 MHz, Bruker, Germany) using DMSO-d_6 and $CDCl_3$ as the solvent and tetramethylsilane (TMS) as the internal standard at 25 °C. UV-vis and CD spectra were recorded on a JASCO J-1500 spectropolarimeter equipped with a Peltier-controlled unit using an SQ-grade cuvette, a single accumulation, a path length of 10 mm, a bandwidth of 2 nm, a scanning rate of 200 nm/min, and a response time of 2 s. The samples were measured at 25 °C unless otherwise declared. The UV-vis and CD data were analyzed using Spectra Manager program. TEM images were taken with a HITACHI HT7700 instrument operated at an accelerating voltage of 120 kV. AFM images were captured with peak force quantitative nanomechanical mapping scan mode on Multimode 8 microscope (Bruker, Germany), AFM samples were prepared by depositing droplets of the solution without dilution onto a silicon wafer until it was fully covered (about 10 μL), followed by slow

evaporation under the poor solvent atmosphere. The thermal behaviors were measured by TA instrument DSC 250 (New Castle, DE, USA). The heating rate and cooling rate were both 10 °C/min. Fourier transform infrared (FT-IR) spectra were recorded on a NICOLET-6700 FT-IR spectrometer with Φ 20 mm × 4 mm potassium bromide tablets. The IR samples were prepared by adding 200 μL solution dropwise and then used Argon purging to evaporate the solvent of the samples. The ESR measurement were performed on a JES-X320 electron spin resonance spectrometer (JEOL, USA) with samples sealed in capillaries.

All assemblies were prepared by adding poor solvent into the monomer solution solvated by good solvent DCE. We employed a standardized solution preparation method for all our samples. Monomer solution dissolved in good solvent was added into the cuvette, then poor solvent was slowly added along the walls of cuvette. Clear stratification between the two solvents could be observed at the bottom of the cuvette. Finally, the cuvette was oscillated ten times and turned over twice to ensure thorough mixing. This preparation method guarantees reproducibility of data and meets qualitative analysis requirements.

Supplemented Figures

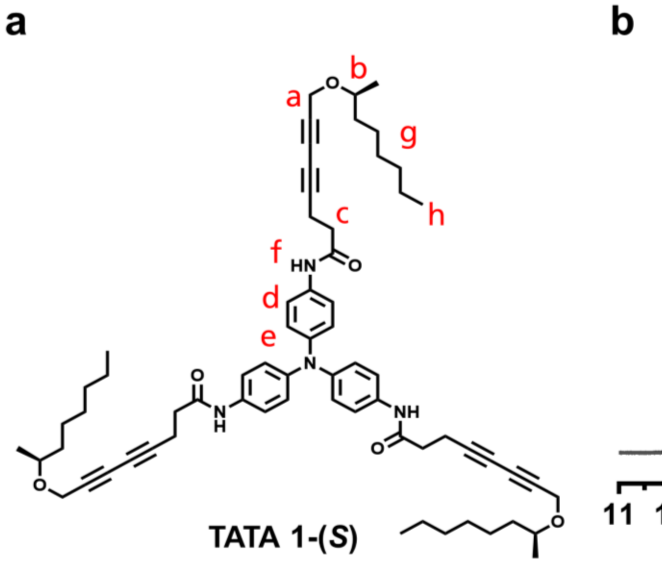

Figure S1. Characterization of the **1*S***.(a) Chemical structure and (b) ^{1}H NMR spectrum of **1*S***.

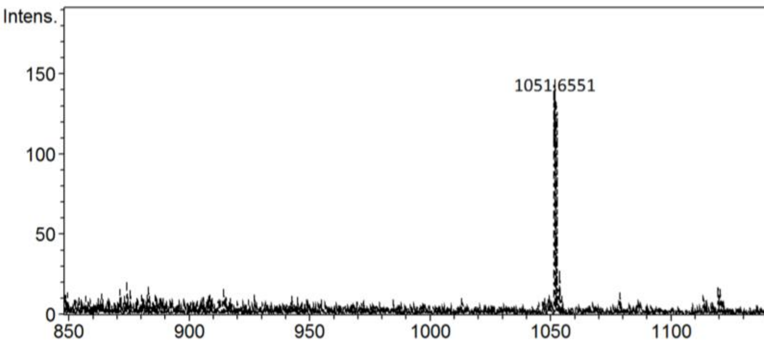

Figure S2. Mass spectroscopy measurement of **1*S*** monomer. Calculated **1*S*** Na^{+}: 1052.41 Da. Measured: 1051.66 Da.

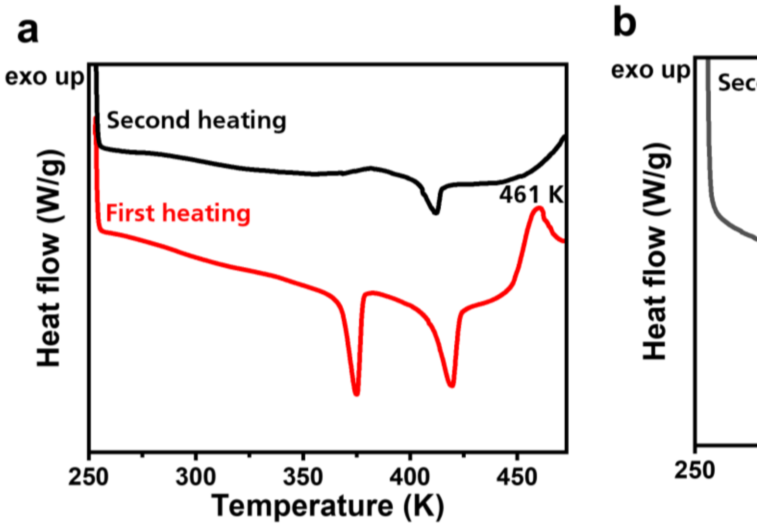

Figure S3. DSC measured curves of **1*S***. (a) DSC measured curves of **1*S***, the heating and cooling rates were both 10 K/min. (b) The order-to-disorder transition is analyzed using TRIOS program in the second heating curve, which is around 300 K (27 °C).

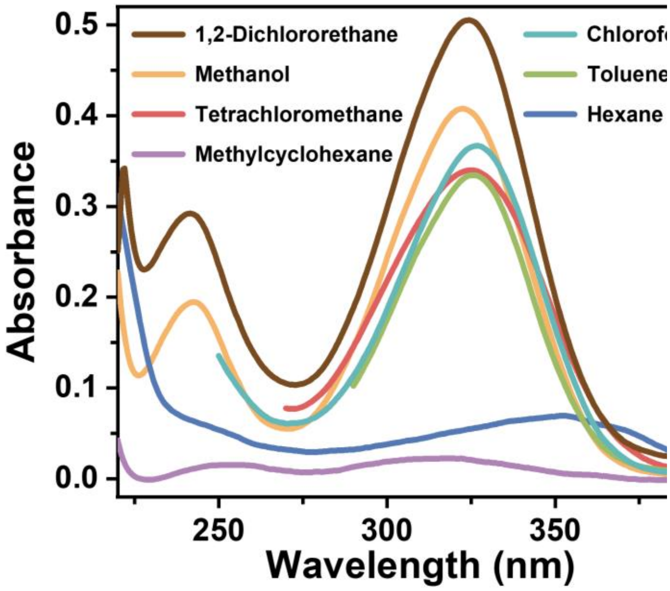

Figure S4. UV-vis spectra of **1*S*** monomer in various single solvents with fixed 20 μM concentration.

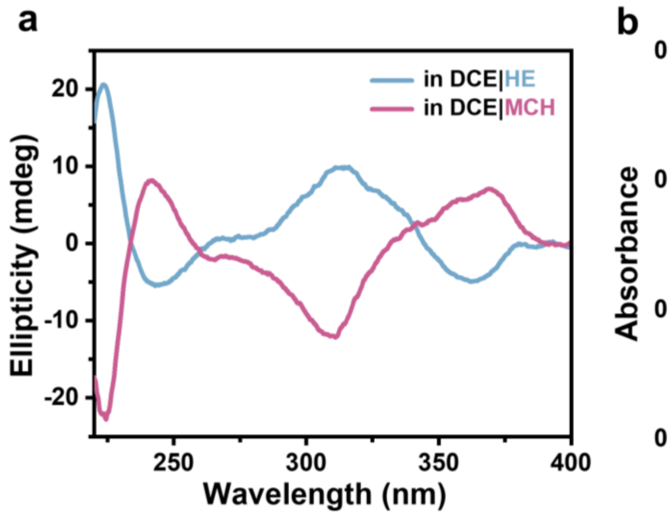

Figure S5. CD and UV spectra of **1*R*** assemblies. (a) CD and (b) UV spectra of **1*R*** assembly in DCE/MCH and DCE/HE (3%, v/v), respectively. The monomer concentration is 32 μM.

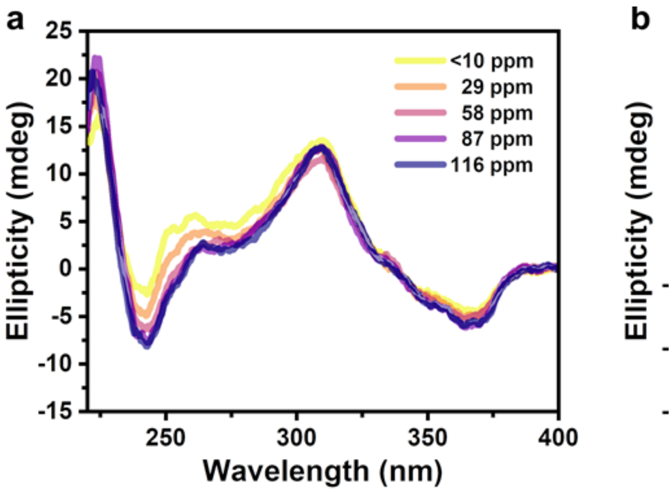

Figure S6. The CD spectra of **1*S*** assembly in (a) DCE/MCH and (b) DCE/HE with various water content in poor solvent. The poor solvents with different water content were prepared by mixing a water-saturated solvent with a drying solvent. The water content was estimated based on the recorded solubility of water in hydrocarbons at 20 ℃[2].

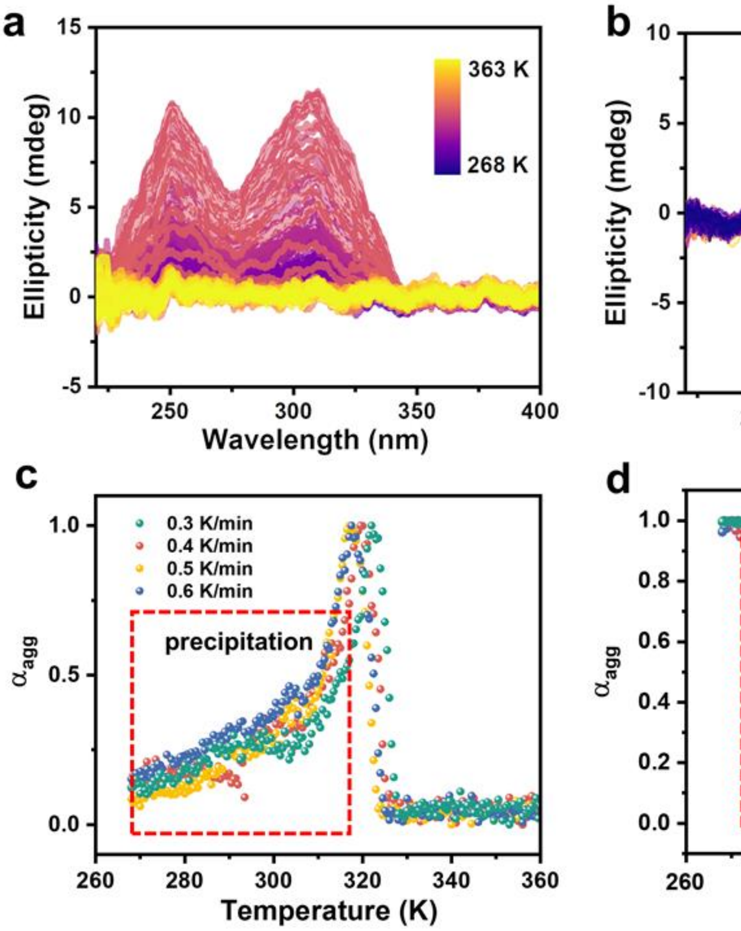

Figure S7. (a) Temperature-dependent CD spectra of **1*S*** assembly in DCE/MCH during a slow cooling process from 363 K to 268 K (cooling rate: 0.3 K/min, concentration: 10 μM). (b) Temperature-dependent CD spectra of **1*S*** assembly in DCE/HE during a slow cooling process from 353 K to 268 K (cooling rate: 0.3 K/min, concentration: 10 μM). (c) The variation in the degree of aggregation (determined by intensities of CD bands) as a function of temperature during the slow cooling process with different cooling rates. (d) The variation in the degree of aggregation (determined by absorbance) as a function of temperature during the slow cooling process with different cooling rates.

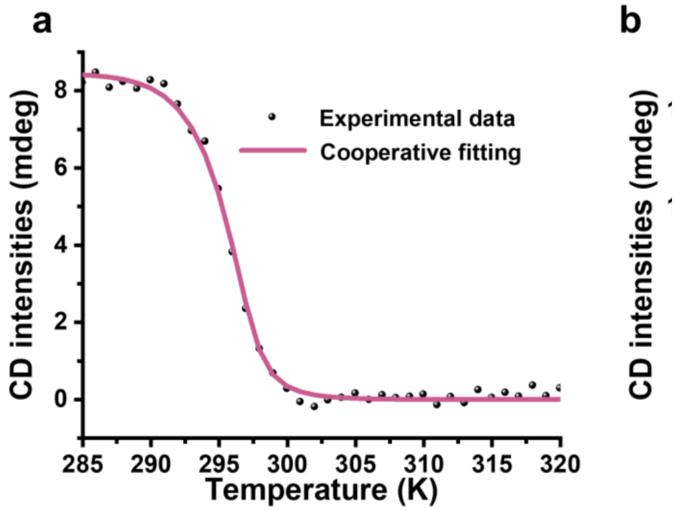

Figure S8. CD spectra of the **1*S*** assemblies in DCE/MCH (3%, v/v) solution with 13.6 μ M and 16.8 μM concentrations. Depolymerization experiments of **1*S*** assemblies in DCE/MCH (3%, v/v) solution by measuring CD intensities at the single wavelength of 311 nm with (a) 13.6 μM and (b) 16.8 μM concentrations. The curves are fitted with mass balance model.

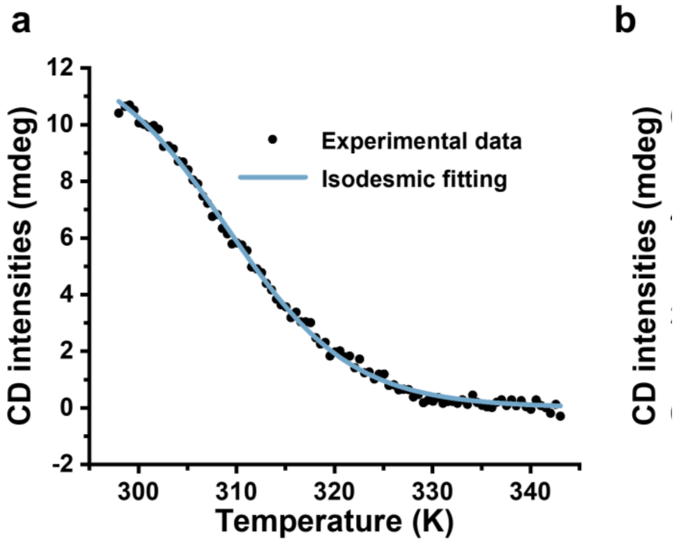

Figure S9. CD spectra of the **1*S*** assemblies in DCE/MCH (3%, v/v) solution with 9.7 μM and 12.9 μM concentrations. Depolymerization experiments of **1*S*** assemblies in DCE/HE (3%, v/v) solution by measuring CD intensities at the single wavelength of 311 nm with (a) 9.7 μM and (b) 12.9 μM concentrations. The curves are fitted with mass balance model.

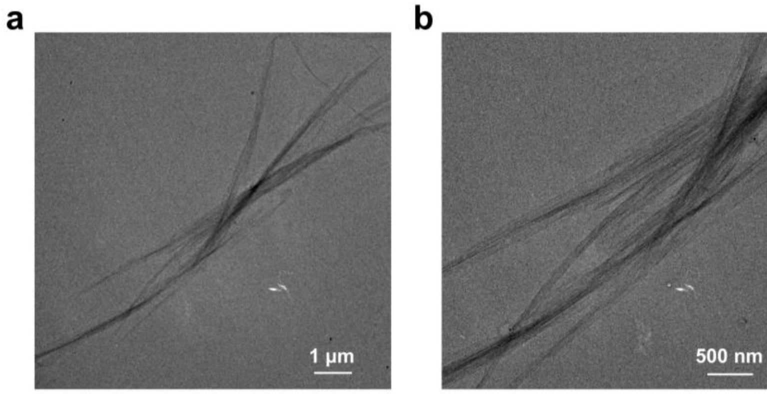

Figure S10. TEM images of **1*S*** aggregates assembled in DCE/MCH (3%, v/v) with 32 μM concentration. The samples were prepared by directly drop-casting assembly solutions onto carbon film supported copper grids.

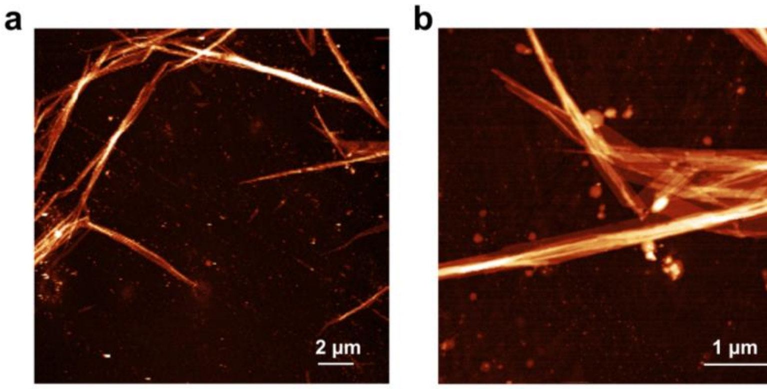

Figure S11. AFM images of **1*S*** aggregates assembled in DCE/MCH (3%, v/v) with 32 μM concentration. The samples were prepared by drop-casting assembly solutions onto freshly cleaned silicon wafers.

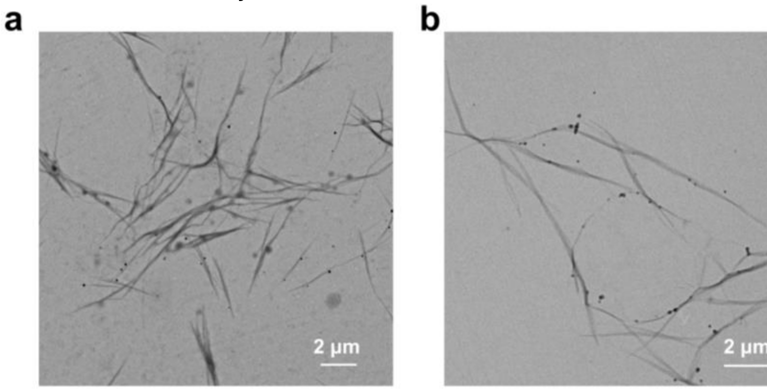

Figure S12. TEM images of **1*S*** aggregates assembled in DCE/HE (3%, v/v) with 32 μM concentration. The samples were prepared by directly drop-casting assembly solutions onto carbon film supported copper grids.

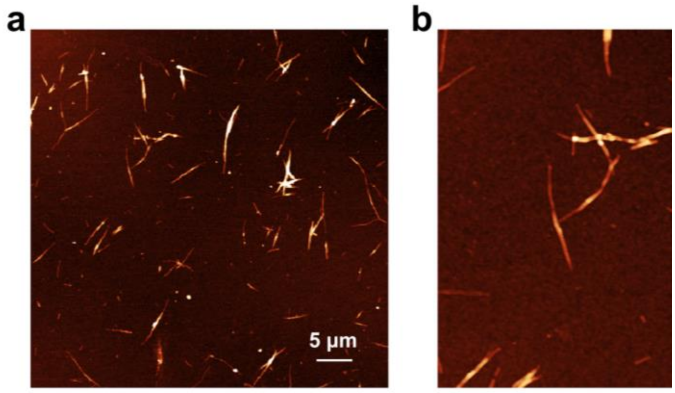

Figure S13. AFM images of **1*S*** aggregates assembled in DCE/HE (3%, v/v) with 32 µM concentration. The samples were prepared by drop-casting assembly solutions onto freshly cleaned silicon wafers.

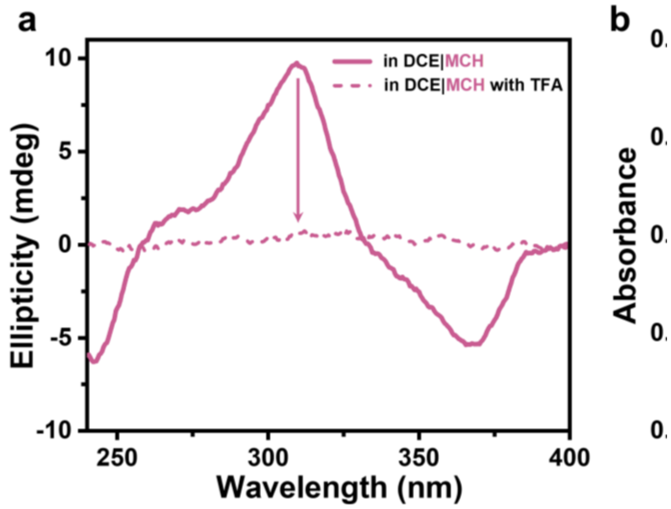

Figure S14. CD and UV spectra of **1*S*** assembly in DCE/MCH before and after adding TFA to dissociate hydrogen bonds. (a) CD and (b) UV spectra. The disappearance of CD signals and increase of absorbance indicate the disassembly of aggregates to loss the entire supramolecular nanostructures.

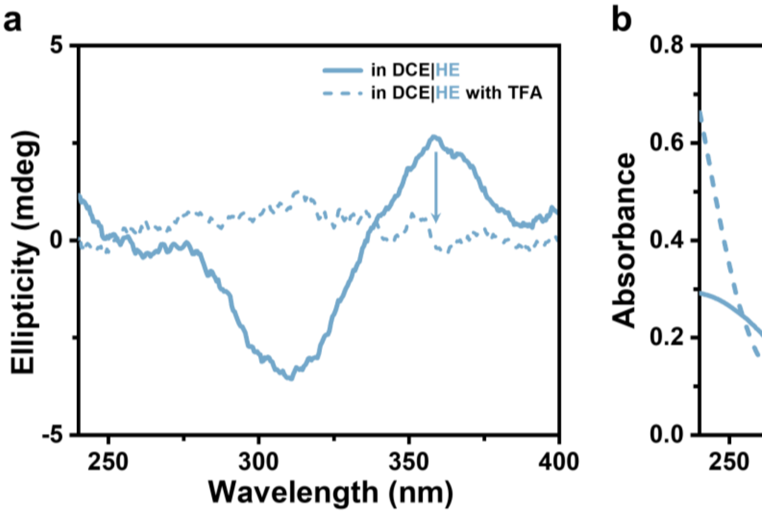

Figure S15. CD and UV spectra of **1*S*** assembly in DCE/HE before and after adding TFA to dissociate hydrogen bonds. (a) CD and (b) UV spectra. Same to that in MCH, the disappearance of CD signals and increase of absorbance indicate the disassembly of aggregates to loss the entire supramolecular nanostructures.

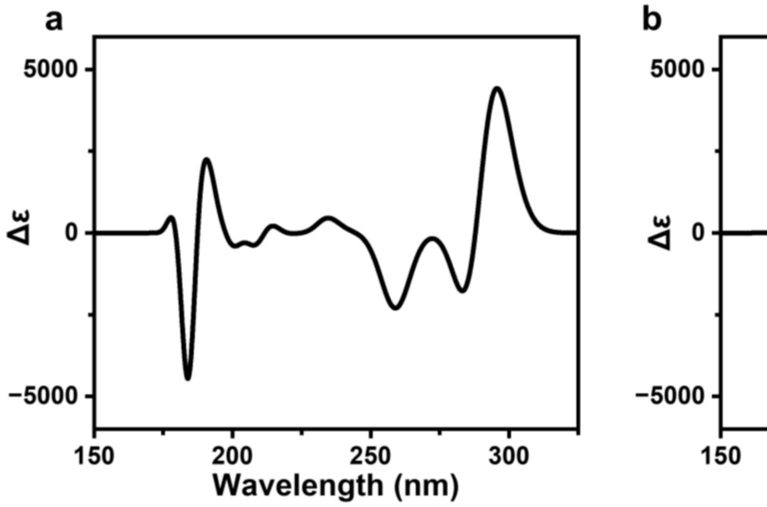

Figure S16. Calculated CD spectra of modified trimers with *P* helical and *M* helical arrangement of central phenyl rings. (a) *P* helical and (b) *M* helical. All calculations were performed using ωB97X-D3 def2-SV(P) with Orca[3-7]. Data visualization was performed using Multiwfn[8] program. Coordinates were provided in appendix.

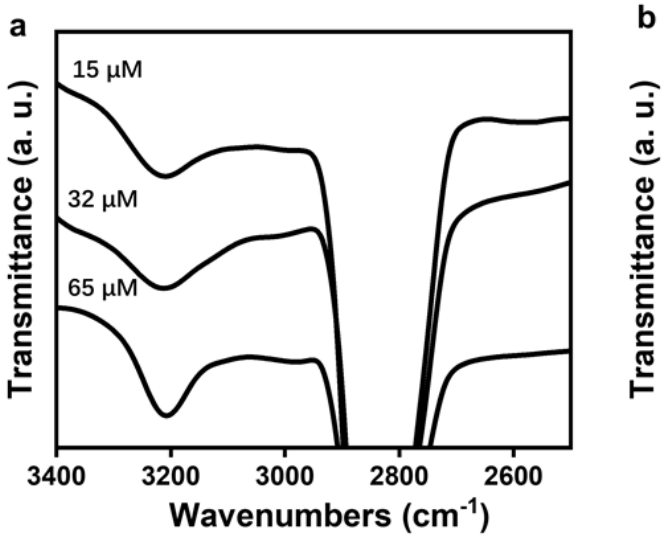

Figure S17. IR spectra of assembly in (a) DCE/MCH and (b) DCE/HE with different concentrations.

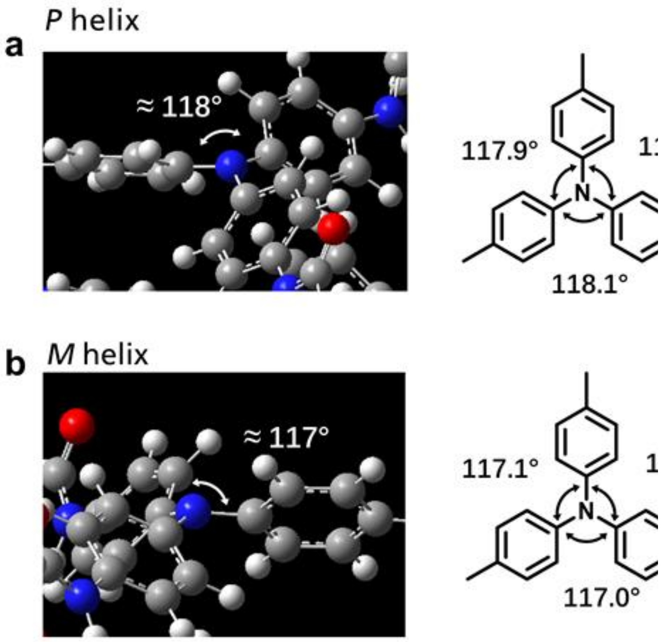

Figure S18. The magnified images of central nitrogen atoms in the modified trimers and the schematic representation of measured C-N-C bond angles in (a) P and (b) M helical structures.

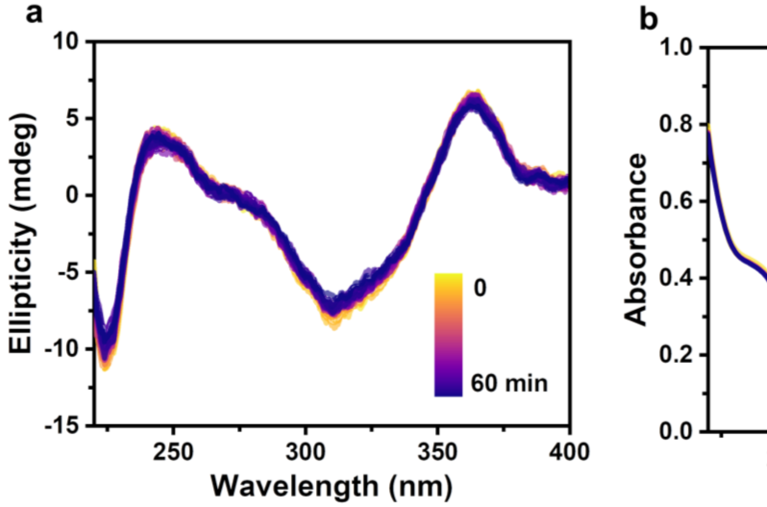

Figure S19. Time-dependent (a) CD and (b) UV spectra of **1*S*** assembly in DCE/HE after addition of MCH (equal amount of HE). The absorbance barely changed within 60 min.

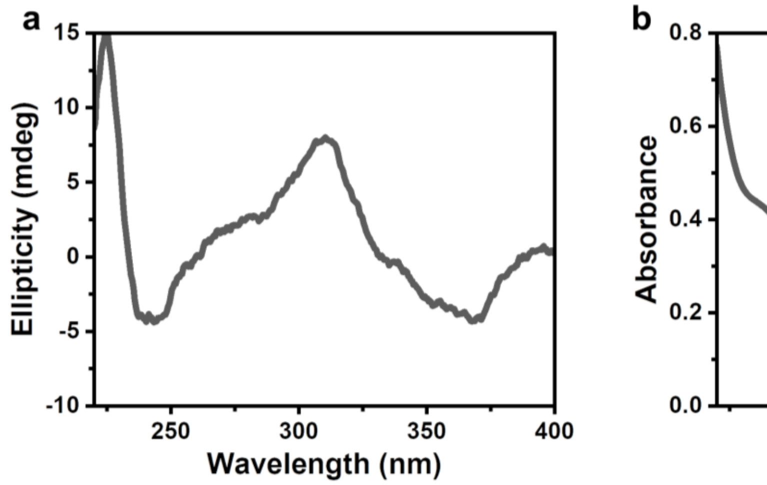

Figure S20. CD and UV spectra of **1*S*** assembly in DCE/MCH. (a) CD and (b) UV spectra of **1*S*** assembly in DCE/MCH prepared with monomer solution irradiated for 30 min (365 nm irradiation wavelength, dark green solution).

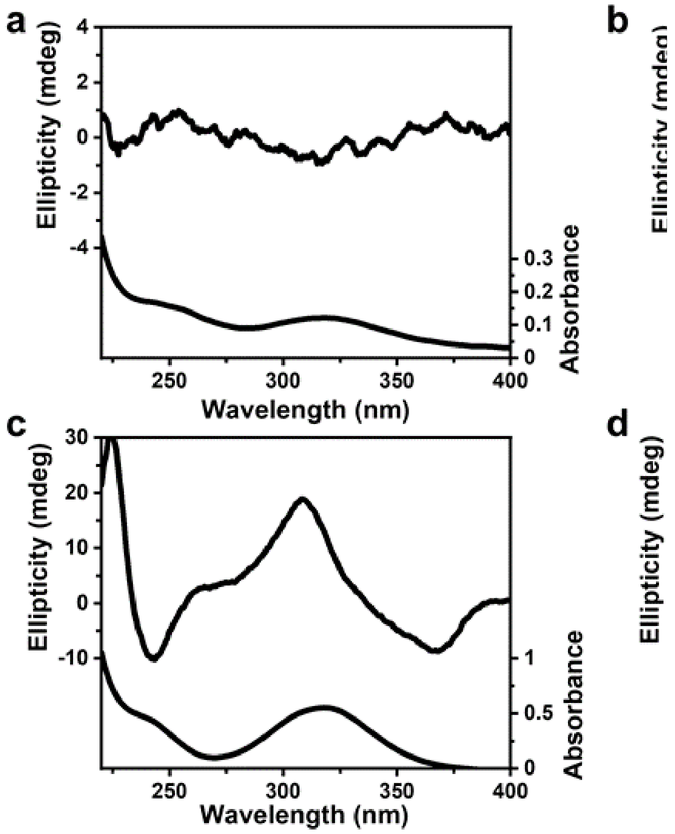

Figure S21. CD spectra of **1*S*** in DCE and various poor solvents. (a) Isooctane. (b) 2-Methylpentane. (c) Decalin. (d) Cyclohexane. c = 32 μM.

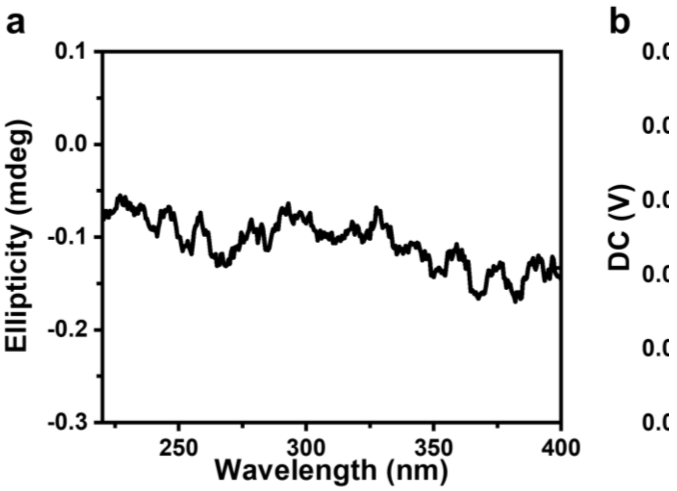

Figure S22. (a) FDCD spectrum and (b) corresponding luminescence of **1*S*** assemblies in DCE/MCH.

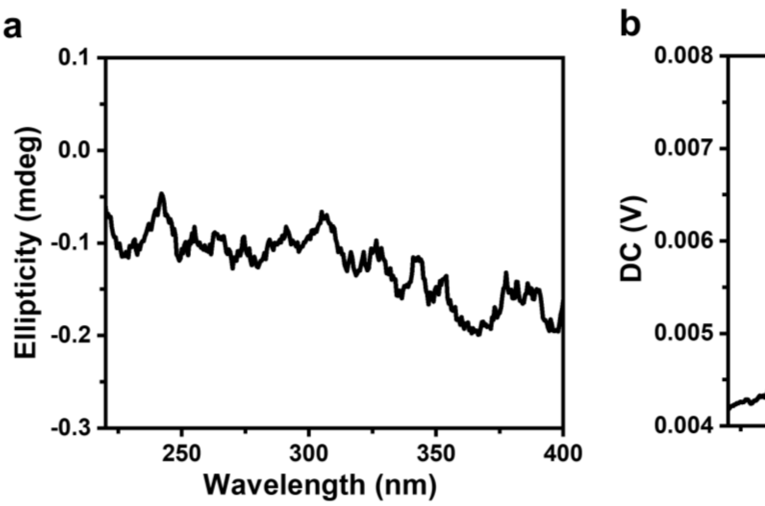

Figure S23. (a) FDCD spectrum and (b) corresponding luminescence of **1*S*** assemblies in DCE/HE.

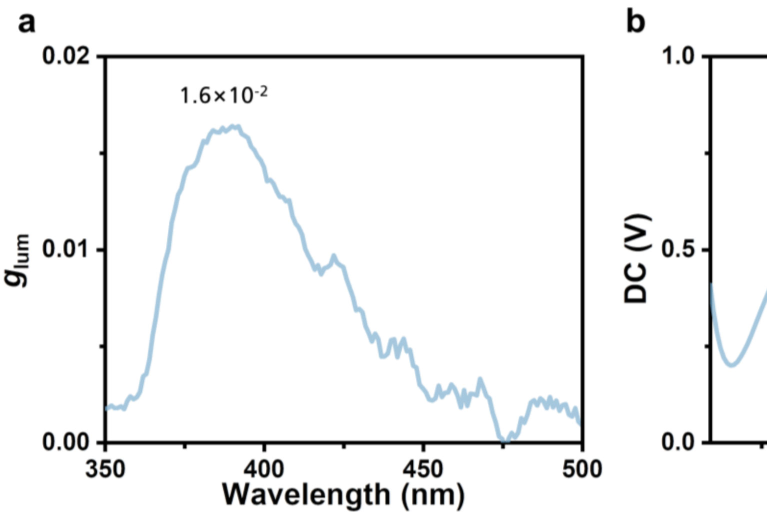

Figure S24. The g_{lum} values and DC spectrum of **1*S*** assemblies in DCE/MCH. (a) The processed g_{lum} values of measured CPL spectrum of **1*S*** assemblies in DCE/MCH. (b) Related luminescence spectrum. The maximum g_{lum} value is 1.6×10^{-2}.

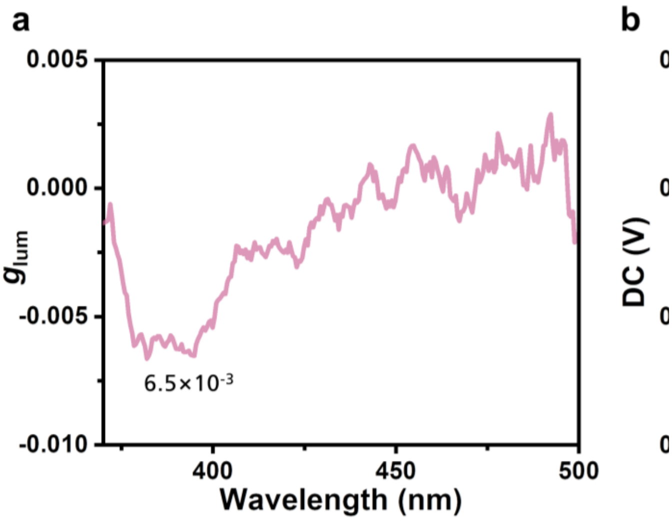

Figure S25. The g_{lum} values and DC spectrum of **1*S*** assemblies in DCE/HE. (a) The processed g_{lum} values of measured CPL spectrum of **1*S*** assemblies in DCE/HE. (b) Related luminescence spectrum. The maximum g_{lum} value is 6.5×10^{-3}.

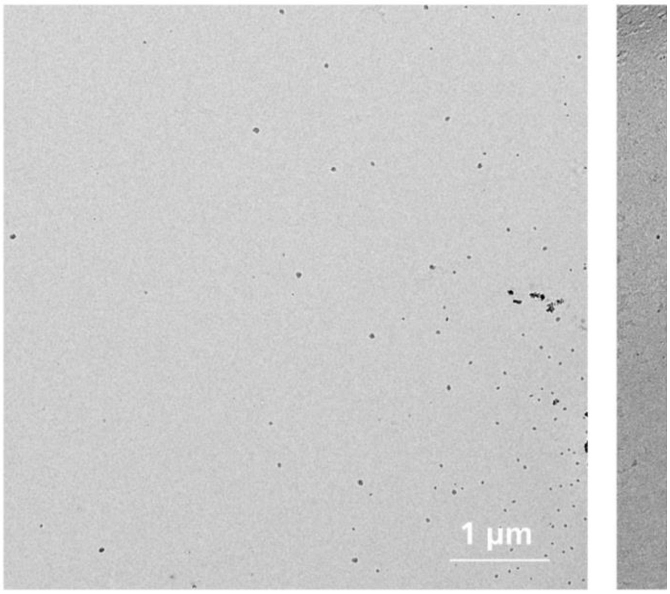

Figure S26. TEM images selected from different areas of **1*S*** in single DCE solvent (c = 32 μM).

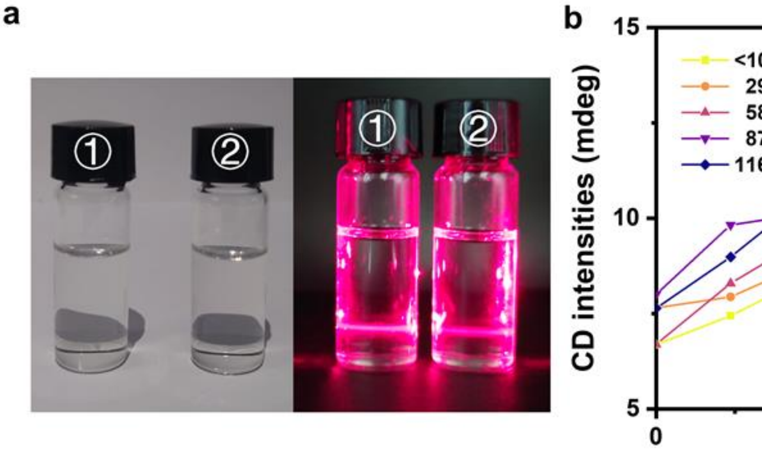

Figure S27. (a) Photos of initial assembly solution (vial 1, right after the addition of poor solvent, c = 32 μM) and the assembly solution after overnight incubation (vial 2) in DCE/MCH. The Tyndall effect was observed in both solutions. (b) Time-dependent CD intensities of assembly (319 nm) in DCE/MCH with different water content in MCH.

References

[1] W. Yin, C. He, M. Chen, H. Zhang, A. Lei, *Org. Lett.* **2009**, *11*, 709-712.

[2] B. A. Énglin, A. F. Platé, V. M. Tugolukov, M. A. Pryanishnikova, *Chem. Technol. Fuels Oils* **1965**, *1*, 722-726.

[3] T. Hočevar, J. Demšar, *J. Stat. Softw.* **2016**, *71*, 1-24.

[4] T. Risthaus, A. Hansen, S. Grimme, *Phys. Chem. Chem. Phys.* **2014**, *16*, 14408-14419.

[5] C. Bannwarth, S. Grimme, *Comput. Theor. Chem.* **2014**, *1040*, 45-53.

[6] S. Grimme, *J. Chem. Phys.* **2013**, *138*, 244104.

[7] U. Ekström, L. Visscher, R. Bast, A. J. Thorvaldsen, K. Ruud, *J. Chem. Theory Comput.* **2010**, *6*, 1971-1980.

[8] T. Lu, F. Chen, *J. Comput. Chem.* **2012**, *33*, 580-592.

Appendix

Cartesian coordinates of modified trimer structure

***P* helix structure**

1	N	0.007487569	0.081393104	-1.008533553
2	C	0.696919191	1.311757812	-0.810747027
3	C	0.177700515	2.322839600	0.010369698
4	C	1.910928754	1.509855511	-1.487792935
5	C	0.878431618	3.508694420	0.174210466
6	H	-0.765098273	2.178081583	0.537164585
7	C	2.618504834	2.690947434	-1.317174917

8	H	2.294410127	0.732958002	-2.151460363
9	C	2.110297070	3.684880846	-0.470940340
10	H	0.479599771	4.290199908	0.818721562
11	H	3.562930607	2.844699578	-1.842259467
12	C	0.731455754	-1.118952368	-0.753755316
13	C	0.285545330	-2.305572154	-1.357975570
14	C	1.875915347	-1.130074686	0.055973860
15	C	0.949957381	-3.499933015	-1.120435536
16	H	-0.584985306	-2.285426877	-2.016360615
17	C	2.546667502	-2.321703471	0.288028983
18	H	2.233898269	-0.212377137	0.521849991
19	C	2.068764078	-3.511282394	-0.278242399
20	H	0.604955795	-4.424135714	-1.587078111
21	H	3.429257020	-2.332200350	0.924717637
22	C	-1.388226941	0.066548417	-0.722189938
23	C	-1.946273900	-0.869242486	0.159977775
24	C	-2.211114772	1.001123010	-1.370466786
25	C	-3.309675084	-0.852593846	0.413113041
26	H	-1.313699238	-1.598607667	0.665128438
27	C	-3.573535259	1.027662658	-1.109285080
28	H	-1.776193751	1.704664415	-2.082669108
29	C	-4.120259499	0.112519606	-0.200937762
30	H	-3.742050544	-1.575298553	1.102671865
31	H	-4.214704985	1.755195561	-1.610413703
32	N	0.103185703	0.083814567	3.343319714
33	C	0.544097283	1.420292573	3.614513347
34	C	-0.089425513	2.228038560	4.568634055
35	C	1.643231851	1.916478908	2.897192134
36	C	0.398331774	3.498759480	4.836123186
37	H	-0.958045573	1.859526015	5.115613752
38	C	2.141867885	3.183918806	3.170598088
39	H	2.111491354	1.297469558	2.128078003
40	C	1.533307219	3.969516799	4.157972594
41	H	-0.096078787	4.119395552	5.581577165
42	H	3.010709008	3.556811481	2.623728988
43	C	1.056654413	-0.944059395	3.634588082
44	C	0.889348706	-2.205965965	3.043197065
45	C	2.152982734	-0.712065808	4.477698817
46	C	1.772403499	-3.239206088	3.332916764
47	H	0.055380169	-2.376048608	2.357980848
48	C	3.045943486	-1.735582174	4.755530111
49	H	2.306098816	0.267960012	4.931748622
50	C	2.843414947	-3.011144404	4.206038898
51	H	1.619840336	-4.225403260	2.888996223
52	H	3.894405987	-1.545372962	5.411232989
53	C	-1.254453961	-0.204487051	3.691743706
54	C	-1.581867960	-1.229206800	4.591055852
55	C	-2.276573259	0.551854954	3.097007244
56	C	-2.907961920	-1.478788084	4.908623660
57	H	-0.799023700	-1.829450276	5.056386407
58	C	-3.606380197	0.314553643	3.424397438
59	H	-2.022150223	1.332520173	2.375852664
60	C	-3.924642507	-0.693251593	4.343127427
61	H	-3.151862156	-2.278772154	5.605986037
62	H	-4.394808832	0.920113350	2.971759587
63	N	-0.062555892	0.000351270	-5.324287769
64	C	0.938683169	0.999397843	-5.187081405
65	C	0.739113010	2.138189105	-4.393018415
66	C	2.132874879	0.846987685	-5.907486753
67	C	1.722258929	3.112029191	-4.326939784
68	H	-0.179171798	2.253890533	-3.817060031
69	C	3.123056425	1.816639347	-5.833616033
70	H	2.282910012	-0.037580900	-6.528253829
71	C	2.922920594	2.954327618	-5.042786450
72	H	1.572612524	3.992671107	-3.700557855
73	H	4.050407123	1.683154562	-6.394342689
74	C	0.315788890	-1.353089977	-5.106473933
75	C	-0.403651922	-2.360174381	-5.767151923
76	C	1.407920805	-1.688100269	-4.293142734
77	C	-0.037579089	-3.690518448	-5.619580125
78	H	-1.251809280	-2.094812045	-6.399892572
79	C	1.781092001	-3.015486673	-4.153068193
80	H	1.957431660	-0.910503874	-3.762681746
81	C	1.059546345	-4.023514395	-4.815466244
82	H	-0.604708539	-4.468669105	-6.134203610
83	H	2.624908842	-3.278065767	-3.514188973
84	C	-1.415143557	0.363317481	-5.089136905
85	C	-2.243494952	-0.397914459	-4.251247009
86	C	-1.933993528	1.481245333	-5.760540105
87	C	-3.576913230	-0.051957755	-4.100487866
88	H	-1.841010243	-1.253910637	-3.709715858
89	C	-3.265921988	1.836014413	-5.600654022
90	H	-1.288430162	2.070494408	-6.413173421
91	C	-4.094818033	1.067358689	-4.774388530
92	H	-4.219634632	-0.640344098	-3.445117493
93	H	-3.660299773	2.709325674	-6.124102940
94	N	-5.483342670	0.172608693	0.185917602
95	H	-5.692076080	-0.099176451	1.149097337
96	N	2.850050185	4.860263124	-0.177902732
97	H	2.744956723	5.237174061	0.765501730
98	N	2.705599760	-4.722976444	0.090919372
99	H	3.080944288	-4.767660720	1.041281168
100	N	-5.247043422	-0.925044320	4.802324250
101	H	-5.323272920	-1.401492171	5.700256512
102	N	2.066351552	5.219136976	4.568429988
103	H	1.827601508	5.515576237	5.512687988
104	N	3.716135436	-4.044110850	4.635715166
105	H	4.203994410	-3.871786405	5.513784436
106	N	3.889603319	3.975021492	-4.888473015
107	H	3.750853073	4.592399435	-4.082025170
108	N	1.479144540	-5.358269549	-4.602988720
109	H	2.023566307	-5.523833352	-3.752342142
110	N	-5.457641548	1.377507018	-4.550951574
111	H	-5.863528359	1.006689122	-3.687779573
112	C	-6.397615901	-0.702091160	4.064659801
113	C	2.752934672	6.089056596	3.738758978
114	C	4.051866939	-5.162438848	3.891563100
115	C	-6.514037052	0.467703151	-0.678450374
116	C	3.617214708	5.528650353	-1.103870409
117	C	2.912822101	-5.776262360	-0.771915757
118	C	-6.249221955	2.101550264	-5.420804343
119	C	5.010259144	4.145841869	-5.678088901
120	C	1.225501108	-6.407060118	-5.465426830
121	O	2.898535362	5.862755895	2.554426500
122	O	-6.365590589	-0.301771262	2.919047416
123	O	3.641579929	-5.333757996	2.761768585
124	O	3.674623818	5.169550233	-2.266973472
125	O	-6.320759491	0.689184461	-1.860423314
126	O	2.580780820	-5.725220568	-1.942663423
127	O	5.262070108	3.430261742	-6.627659625

128	O	-5.817218686	2.577399767	-6.453033547
129	O	0.574207427	-6.270874192	-6.483034698
130	C	3.301483487	7.337729108	4.391195330
131	H	3.692432667	8.014345690	3.594545368
132	H	2.490423729	7.910626270	4.884396608
133	C	-7.695969703	-0.989375384	4.785212451
134	H	-8.524651844	-1.004662376	4.038401922
135	H	-7.689998607	-2.008129326	5.221488684
136	C	4.957642802	-6.156719691	4.582581545
137	H	5.345786848	-6.876113876	3.823271095
138	H	5.860570788	-5.656793646	4.986303157
139	C	4.409090535	6.715298575	-0.604673220
140	H	4.972692868	7.152357771	-1.461338306
141	H	3.734515920	7.525214704	-0.261281831
142	C	-7.899337231	0.514006466	-0.077320120
143	H	-8.639178838	0.643297416	-0.900755334
144	H	-8.158357516	-0.455492032	0.393488636
145	C	3.558600570	-7.007887645	-0.180770810
146	H	3.804367015	-7.713376086	-1.006976494
147	H	4.531788292	-6.761087285	0.289031289
148	C	5.895429905	5.317891652	-5.306469913
149	H	6.678191745	5.431188917	-6.090885672
150	H	5.318366918	6.265557573	-5.330934627
151	C	-7.708279699	2.227982484	-5.036986439
152	H	-8.243354838	2.739416195	-5.870659200
153	H	-8.174929255	1.223296221	-4.964977351
154	C	1.849836426	-7.735292832	-5.093815980
155	H	1.639780329	-8.459011486	-5.915624920
156	H	2.955763623	-7.644133591	-5.062957700
157	C	4.419651668	7.012110388	5.394177078
158	H	4.567793620	7.891598593	6.073542337
159	H	4.112603754	6.182623576	6.076703170
160	C	-7.977138752	0.065100067	5.869927212
161	H	-8.482946086	-0.424638647	6.741333735
162	H	-7.024378306	0.467746731	6.294997642
163	C	4.209925604	-6.910735670	5.696099905
164	H	4.914042503	-7.113727941	6.543625610
165	H	3.412113889	-6.270444777	6.148063996
166	C	5.370472354	6.293346989	0.517957602
167	H	4.867684072	6.392268761	1.516011905
168	H	5.612422217	5.201062887	0.441324891
169	C	-8.012562878	1.659819320	0.945151544
170	H	-7.914152745	1.256600623	1.986036531
171	H	-7.150896912	2.369658645	0.848420266
172	C	2.616229937	-7.668323351	0.842674407
173	H	2.926319181	-7.388913570	1.882804036
174	H	1.576306932	-7.260953927	0.751589298
175	C	6.549805316	5.117793985	-3.929544166
176	H	5.777342392	5.167639715	-3.117535913
177	H	6.981044867	4.088533097	-3.852306179
178	C	-7.903946596	3.007637797	-3.726033737
179	H	-7.595238861	2.382316217	-2.847852863
180	H	-7.225762891	3.896897669	-3.696016925
181	C	1.317243378	-8.282120029	-3.758795159
182	H	1.747678260	-7.705763694	-2.898540277
183	H	0.212342910	-8.122242772	-3.684708334
184	C	5.652442839	6.693597971	4.739258924
185	C	-8.793832144	1.129965265	5.372505924
186	C	3.655064321	-8.142334997	5.223185522
187	C	6.578291774	7.055237947	0.480479188

188	C	-9.244701560	2.363185638	0.774812843
189	C	2.599996041	-9.086561596	0.670864615
190	C	7.560763737	6.109527414	-3.729148519
191	C	-9.273136961	3.409397502	-3.617989053
192	C	1.636887173	-9.673564591	-3.659848369
193	C	6.715350729	6.439559927	4.206290722
194	C	-9.514145865	2.024158582	4.972952299
195	C	3.204184409	-9.206942708	4.846578301
196	C	7.629400105	7.665775065	0.450409445
197	C	-10.283770639	2.969654795	0.598907912
198	C	2.573048632	-10.289850005	0.499072687
199	C	8.419280950	6.961338092	-3.601131034
200	C	-10.443093488	3.741140655	-3.587602558
201	C	1.917028243	-10.857157310	-3.638643473
202	C	7.912852274	6.179623119	3.624370355
203	C	-10.347138269	3.004025842	4.542983620
204	C	2.730780949	-10.411244737	4.441261758
205	C	8.838981503	8.276276228	0.428712485
206	C	-11.418055860	3.681696439	0.392514246
207	C	2.502994437	-11.627961874	0.297569805
208	C	9.378191526	7.913565229	-3.492356879
209	C	-11.749239733	4.105011708	-3.600125232
210	C	2.235639398	-12.175065182	-3.662248588
211	C	8.988948956	5.966052818	3.099773053
212	C	-11.126302386	3.858712113	4.168132865
213	C	2.342279945	-11.507680553	4.087021572
214	C	9.947016376	8.775478988	0.422162250
215	C	-12.425129751	4.336715833	0.208058691
216	C	2.423171205	-12.827101328	0.116520787
217	C	10.234403382	8.772523975	-3.413683624
218	C	-12.923214547	4.418312803	-3.638904840
219	C	2.535416316	-13.352243630	-3.711782229
220	C	10.268041593	5.731587262	2.486279327
221	H	10.382017578	4.682498561	2.130177003
222	H	10.439277892	6.418659071	1.621079204
223	C	-12.069919774	4.841759908	3.713782912
224	H	-12.434900800	4.605048120	2.684581706
225	H	-12.947482316	4.918192918	4.395843615
226	C	1.918968875	-12.810518342	3.654686523
227	H	2.250602362	-13.014768557	2.607273396
228	H	2.312005893	-13.613950410	4.318949776
229	C	11.294868670	9.271227408	0.399829079
230	H	11.438113693	10.069776289	-0.359994429
231	H	11.619140095	9.662559892	1.390552988
232	C	-13.569648791	5.173457772	-0.020391772
233	H	-14.101398181	4.897294819	-0.958437179
234	H	-14.296948371	5.143547517	0.820768657
235	C	2.254832279	-14.235377213	-0.107802226
236	H	2.722081688	-14.558232624	-1.065302256
237	H	2.673777774	-14.853895629	0.716286607
238	C	11.254664448	9.779367100	-3.335599746
239	H	10.833197149	10.794361688	-3.151869307
240	H	11.992566979	9.545411667	-2.533986070
241	C	-14.307734250	4.790361401	-3.716646373
242	H	-14.983045660	3.903798400	-3.694767679
243	H	-14.597277689	5.478403817	-2.891035998
244	C	2.889140116	-14.740867755	-3.802958089
245	H	3.993428827	-14.892957115	-3.820400033
246	H	2.462944184	-15.332762184	-2.962315661
247	O	-11.378373339	6.088034771	3.686404683

248	O	11.237035180	5.940722364	3.511648113
249	O	0.494233866	-12.813192446	3.700700292
250	O	-13.034788192	6.493068174	-0.120257168
251	O	12.086482234	8.121541237	0.096778801
252	O	0.840951594	-14.425200045	-0.155262426
253	O	-14.479771972	5.419875883	-4.986475985
254	O	11.886038149	9.804348863	-4.615645554
255	O	2.381384416	-15.200589869	-5.055555427
256	C	-12.186900762	7.169807750	3.217981156
257	H	-12.894557636	6.810855452	2.436498698
258	C	12.507345003	6.378531922	3.033255400
259	H	12.358943476	7.235699016	2.335664279
260	C	-0.081638550	-14.039025366	3.242862024
261	H	0.539422220	-14.476216284	2.428435968
262	C	-13.940058968	7.464243536	-0.634555189
263	H	-14.573591708	7.022422716	-1.433243778
264	C	13.369130829	8.398050973	-0.455469901
265	H	13.295165635	9.216694402	-1.205263899
266	C	0.428991022	-15.696096022	-0.647808830
267	H	1.101391055	-16.034014627	-1.465248511
268	C	-15.596838824	6.304122940	-5.047973017
269	H	-16.442446245	5.884480772	-4.461466438
270	C	13.195744600	10.368585417	-4.608108065
271	H	13.242967798	11.209498050	-3.883148196
272	C	2.159264257	-16.608357714	-5.107713144
273	H	2.960138488	-17.137089025	-4.547052697
274	C	13.213594153	6.823131806	4.326768906
275	H	12.480795085	7.331404479	4.985844372
276	H	13.557199482	5.940945644	4.896619119
277	C	-12.928329095	7.763679467	4.427698688
278	H	-13.250379091	6.947858867	5.103067820
279	H	-12.234087851	8.377427819	5.031492126
280	C	-0.189239310	-14.988518566	4.448114236
281	H	0.719297617	-14.898610411	5.073929852
282	H	-1.021499241	-14.670692302	5.103730114
283	C	14.325061250	8.778258910	0.682612031
284	H	13.821630734	9.486246916	1.368977907
285	H	14.550200933	7.881218165	1.291306360
286	C	-14.798478137	7.986309434	0.527340982
287	H	-15.141038963	7.137075943	1.148482187
288	H	-14.174722143	8.610801856	1.195778999
289	C	0.436004434	-16.690626783	0.522630541
290	H	1.358868141	-16.558644753	1.118809017
291	H	-0.398526586	-16.455963606	1.211378656
292	C	14.205592035	9.264603684	-4.253937765
293	H	13.772895408	8.591471284	-3.488648290
294	H	14.381181708	8.614738516	-5.131910982
295	C	-15.169715038	7.681141010	-4.512962803
296	H	-14.423312435	7.556830746	-3.704158997
297	H	-14.636630696	8.246605321	-5.300410254
298	C	0.769091297	-16.912770529	-4.525092255
299	H	0.533078389	-16.193866738	-3.716324049
300	H	-0.011032353	-16.738009790	-5.289944022
301	C	13.225623976	5.220479139	2.343206380
302	H	12.763845662	4.978784643	1.379155813
303	H	14.278138446	5.458341523	2.156975207
304	H	13.193034944	4.314108874	2.960092870
305	C	-11.170156035	8.137119330	2.615731406
306	H	-10.724209783	7.712620780	1.705591625
307	H	-11.638771990	9.089543205	2.349672002
308	H	-10.350046104	8.341081568	3.314919501
309	C	-1.445250387	-13.609882198	2.705584688
310	H	-1.329322361	-12.994748456	1.802484919
311	H	-2.060381815	-14.477070680	2.446798382
312	H	-1.992594700	-13.007475621	3.440815497
313	C	13.736414027	7.069873830	-1.116213743
314	H	13.041168055	6.828177586	-1.931333061
315	H	14.751445917	7.092554934	-1.524429377
316	H	13.681712793	6.241526466	-0.397205608
317	C	-13.001710608	8.531687704	-1.197383976
318	H	-12.475263518	8.166120819	-2.088095422
319	H	-13.543321396	9.442976781	-1.466839994
320	H	-12.230859200	8.802902244	-0.462261048
321	C	-0.979889851	-15.414101711	-1.169377747
322	H	-0.950805888	-14.776297892	-2.061966901
323	H	-1.508800540	-16.337589647	-1.422274685
324	H	-1.576924458	-14.879777421	-0.417129541
325	C	13.365932836	10.869600039	-6.041963015
326	H	12.676991567	11.694258815	-6.259213828
327	H	14.386936287	11.219781480	-6.222472617
328	H	13.149037243	10.073031538	-6.766859108
329	C	-15.930040573	6.340694411	-6.539031399
330	H	-16.287791860	5.365657887	-6.890248195
331	H	-16.701177859	7.086761478	-6.755209906
332	H	-15.042132380	6.588902433	-7.136663590
333	C	2.241045027	-16.919360898	-6.601630171
334	H	3.252032197	-16.746684417	-6.989112105
335	H	1.968768593	-17.959604428	-6.805759251
336	H	1.564471582	-16.273268364	-7.177628673
337	C	14.382234789	7.756793065	4.007124798
338	H	15.082418350	7.260671111	3.306698024
339	H	14.010612657	8.661120565	3.486794541
340	C	-14.136659583	8.593583766	3.988668090
341	H	-13.811072711	9.421603508	3.330068809
342	H	-14.827175045	7.973862115	3.384957606
343	C	-0.380674278	-16.440039014	4.002461493
344	H	-1.286651451	-16.528946357	3.372594423
345	H	0.467880465	-16.760719768	3.368100316
346	C	15.620150810	9.397199876	0.155920364
347	H	16.168568418	8.667230277	-0.470653529
348	H	15.401745260	10.256070219	-0.505877584
349	C	-16.000302103	8.784105366	0.020879243
350	H	-15.659839275	9.648734517	-0.582217502
351	H	-16.616714259	8.168475084	-0.660514442
352	C	0.330815900	-18.134406302	0.031131349
353	H	-0.599883244	-18.273845005	-0.553337583
354	H	1.158549339	-18.370171455	-0.663512674
355	C	15.521934727	9.867145079	-3.757005642
356	H	15.950515170	10.535424301	-4.528197657
357	H	15.340815435	10.506065980	-2.871827245
358	C	-16.380920198	8.469788752	-4.009008598
359	H	-17.149418130	8.537793484	-4.802498492
360	H	-16.862343147	7.935897697	-3.168002010
361	C	0.699624738	-18.350012255	-4.003287803
362	H	0.996442472	-19.059558898	-4.799005382
363	H	1.427247384	-18.495547753	-3.182535631
364	C	15.135195675	8.170436599	5.277413799
365	H	14.450238879	8.683731529	5.977363779
366	H	15.500957273	7.275702984	5.814309052
367	C	-14.879776184	9.156994493	5.206672268

368	H	-15.187513922	8.333060535	5.877309972
369	H	-14.201214283	9.794172781	5.804222019
370	C	-0.498136998	-17.368970187	5.217692378
371	H	0.396654852	-17.268638216	5.860026799
372	H	-1.355847149	-17.063756961	5.846137761
373	C	16.500643109	9.847785687	1.327682481
374	H	15.981191453	10.627068356	1.916372880
375	H	16.665160364	9.002038208	2.023220332
376	C	-16.849920455	9.274034568	1.199214850
377	H	-17.250305927	8.413339574	1.766176650
378	H	-16.216859015	9.839028417	1.910847788
379	C	0.348145430	-19.103462535	1.218893032
380	H	1.300432469	-19.012563982	1.773346959
381	H	-0.449498338	-18.831531692	1.937441673
382	C	16.528172640	8.763583295	-3.407504332
383	H	16.095276950	8.080139226	-2.652563561
384	H	16.730867117	8.137089381	-4.296592802
385	C	-15.965141455	9.877856101	-3.565416817
386	H	-15.170715907	9.814526055	-2.797843871
387	H	-15.518357526	10.427154235	-4.415655424
388	C	-0.715385959	-18.680864255	-3.513026906
389	H	-1.025350733	-17.958363720	-2.734487974
390	H	-1.441579697	-18.561624858	-4.339350769
391	C	16.310375728	9.087491839	4.914287697
392	H	15.944371991	9.969844323	4.356124621
393	H	17.002494503	8.561728228	4.229302415
394	C	-16.107824393	9.963310383	4.767104205
395	H	-16.777084841	9.332749026	4.151865516
396	H	-15.798537495	10.802821392	4.116331096
397	C	-0.664617964	-18.827408549	4.774101644
398	H	0.200141321	-19.139384871	4.158515842
399	H	-1.553313548	-18.928353490	4.122977996
400	C	17.846888550	10.377539674	0.825200636
401	H	17.690114793	11.203767423	0.107344276
402	H	18.376967240	9.584733664	0.262199275
403	C	-17.996678597	10.161453913	0.705147312
404	H	-18.631976032	9.604578368	-0.008101983
405	H	-17.588344408	11.021314068	0.138533825
406	C	0.149319052	-20.545508976	0.742134394
407	H	0.946560043	-20.826570414	0.029537876
408	H	-0.801464934	-20.626978751	0.179530711
409	C	17.835206445	9.370295337	-2.882579074
410	H	17.622954529	10.097816496	-2.076262690
411	H	18.332696037	9.948593069	-3.683179184
412	C	-17.170554850	10.650535979	-3.016593671
413	H	-17.930831841	10.780158147	-3.809217211
414	H	-17.667451791	10.067735103	-2.218129431
415	C	-0.775935815	-20.110702776	-2.962656391
416	H	-0.003632436	-20.253644769	-2.183282990
417	H	-0.531845355	-20.835178611	-3.761795745
418	C	17.067747585	9.543643412	6.167120324
419	H	17.419780610	8.664249813	6.738903333
420	H	16.385164532	10.090190240	6.844801146
421	C	-16.874055607	10.499748512	5.983032553
422	H	-17.186014622	9.661291039	6.634081579
423	H	-16.208216624	11.129925415	6.602652496
424	C	-0.799663263	-19.755412443	5.988282161
425	H	-1.666497847	-19.448847872	6.604104124
426	H	0.085992894	-19.653265386	6.643524130
427	C	18.714990013	10.857691191	1.994780703
428	H	18.847616903	10.040412410	2.729466529
429	H	18.199737752	11.670416031	2.540968519
430	C	-18.844305966	10.666708381	1.878682401
431	H	-19.291936265	9.811618142	2.419144356
432	H	-18.200261042	11.184592986	2.615857740
433	C	0.137443103	-21.518447211	1.927019675
434	H	-0.635963268	-21.214037913	2.659009346
435	H	1.100576042	-21.468732655	2.468862968
436	C	18.775848746	8.276238674	-2.360818554
437	H	18.307903883	7.750272617	-1.506802109
438	H	18.931546409	7.505929591	-3.140229603
439	C	-16.740375843	12.018837470	-2.471621420
440	H	-16.165375477	12.569691498	-3.240440525
441	H	-16.049521164	11.886183191	-1.617115833
442	C	-2.163528662	-20.415532818	-2.383442218
443	H	-2.372393933	-19.746027021	-1.527276752
444	H	-2.945504510	-20.195192289	-3.135208741
445	C	18.253457332	10.431674410	5.789565652
446	H	17.931365097	11.314897079	5.226355315
447	H	18.785905261	10.785640835	6.679354619
448	H	18.976694261	9.892026521	5.167496025
449	C	-18.097425673	11.304033666	5.544663710
450	H	-18.791721207	10.691309742	4.958267397
451	H	-18.650598350	11.690728916	6.407893273
452	H	-17.812539669	12.162715638	4.925837579
453	C	-0.959324588	-21.210306398	5.548492249
454	H	-0.088545098	-21.555994413	4.979530324
455	H	-1.072912977	-21.876997974	6.410665884
456	H	-1.842429491	-21.343052588	4.913031393
457	C	20.076465543	11.339964425	1.496263749
458	H	19.971835387	12.147071781	0.762389765
459	H	20.690349932	11.721758448	2.319738311
460	H	20.638081687	10.530064203	1.016871947
461	C	-19.940782273	11.611629768	1.388819234
462	H	-20.596104362	11.122126137	0.659654722
463	H	-20.569558810	11.956407502	2.217348484
464	H	-19.518258698	12.500813700	0.906818737
465	C	-0.126609727	-22.946937385	1.453017081
466	H	0.623610410	-23.273213768	0.723918924
467	H	-0.103224912	-23.655153888	2.288737285
468	H	-1.109440550	-23.035723756	0.975957203
469	C	20.120074611	8.868143954	-1.937978611
470	H	19.998086205	9.643787607	-1.172808198
471	H	20.780644554	8.099014843	-1.522282539
472	H	20.640951539	9.326954352	-2.786164108
473	C	-17.954795577	12.841325608	-2.042073894
474	H	-18.560458336	12.310421742	-1.298179115
475	H	-17.652300376	13.796064030	-1.597510428
476	H	-18.608249346	13.066026303	-2.892740953
477	C	-2.261951633	-21.875791764	-1.943187380
478	H	-1.486007653	-22.131126536	-1.211787230
479	H	-3.232020581	-22.087551772	-1.479731856
480	H	-2.145556426	-22.559174071	-2.791973831

***M* helix structure**

1	N	0.002445693	-0.000732352	-2.247761415
2	C	0.209061778	1.389247312	-2.010337766
3	C	-0.604440719	2.306046012	-2.694006659
4	C	1.212357494	1.852830254	-1.147212342
5	C	-0.433826282	3.670877189	-2.503513263
6	H	-1.369695493	1.941701293	-3.382501874

7	C	1.390352474	3.216194558	-0.961838157
8	H	1.845243400	1.146550544	-0.609588721
9	C	0.561536197	4.129491774	-1.631823174
10	H	-1.072557182	4.377072219	-3.035749849
11	H	2.162845742	3.574009473	-0.280951575
12	C	1.097202413	-0.878245664	-2.001764145
13	C	0.981387644	-1.974110152	-1.134132498
14	C	2.299279569	-0.651456403	-2.689011255
15	C	2.058437702	-2.828483893	-0.952443484
16	H	0.051702607	-2.154366255	-0.594611671
17	C	3.382584079	-1.499066793	-2.498874671
18	H	2.376565128	0.187105635	-3.383790012
19	C	3.265525551	-2.588711023	-1.627755084
20	H	1.969905534	-3.676698745	-0.273285860
21	H	4.316320312	-1.316314000	-3.033513355
22	C	-1.305184736	-0.514469956	-2.008040758
23	C	-1.703219805	-1.668202386	-2.700685333
24	C	-2.196922963	0.120215652	-1.131668629
25	C	-2.975022869	-2.192230657	-2.512035842
26	H	-1.011957303	-2.149422930	-3.394815221
27	C	-3.470551313	-0.396545518	-0.949330744
28	H	-1.890774446	1.010711230	-0.583949242
29	C	-3.861164453	-1.559340950	-1.632144028
30	H	-3.278751831	-3.090612669	-3.052357874
31	H	-4.160613474	0.091920449	-0.261439750
32	N	0.015048505	0.024356644	2.449655633
33	C	0.626475445	1.288101726	2.692121568
34	C	0.128027537	2.405749326	2.005565172
35	C	1.720935739	1.425805888	3.556903675
36	C	0.705024744	3.653247473	2.197332388
37	H	-0.709178558	2.286690271	1.314127037
38	C	2.309482680	2.669358928	3.736852469
39	H	2.112669563	0.560214042	4.091564031
40	C	1.800007129	3.788434430	3.061427797
41	H	0.300765549	4.520158646	1.669878273
42	H	3.163456523	2.768216923	4.405689064
43	C	0.807810293	-1.133975865	2.698808941
44	C	0.399914372	-2.131813268	3.594002505
45	C	2.012463330	-1.272407929	1.992572439
46	C	1.191407291	-3.255359573	3.786244763
47	H	-0.536458489	-2.028689883	4.143719192
48	C	2.811588976	-2.388865417	2.194028670
49	H	2.314507870	-0.501458204	1.279525351
50	C	2.403883287	-3.383879375	3.092804694
51	H	0.865120706	-4.030701158	4.478448216
52	H	3.752078774	-2.484145450	1.646139032
53	C	-1.383935811	-0.082670015	2.698672772
54	C	-2.100998364	-1.074054539	2.010952410
55	C	-2.051359321	0.786771048	3.572228704
56	C	-3.467680281	-1.208328649	2.209774469
57	H	-1.579227312	-1.733832990	1.313969401
58	C	-3.420867415	0.663602961	3.760522160
59	H	-1.499312822	1.559184587	4.109019232
60	C	-4.132223982	-0.338783627	3.084976780
61	H	-4.015774356	-1.990750279	1.680009691
62	H	-3.934057231	1.346613648	4.436789004
63	N	-0.005467675	0.000876067	-6.909490503
64	C	-0.199609765	1.393022571	-6.670926523
65	C	-1.199777138	2.053011648	-7.400509861
66	C	0.606713647	2.108604668	-5.774864720
67	C	-1.396353425	3.418040432	-7.239440738
68	H	-1.822527576	1.489229428	-8.096661575
69	C	0.414526277	3.472026005	-5.615616102
70	H	1.375532700	1.596496180	-5.196222613
71	C	-0.591043751	4.131295887	-6.343593125
72	H	-2.172850494	3.928484015	-7.812354055
73	H	1.034339132	4.030407332	-4.912505404
74	C	1.295021775	-0.526996648	-6.660310562
75	C	1.503322542	-1.584992857	-5.764482574
76	C	2.372978285	0.008859518	-7.380576850
77	C	2.777017500	-2.106503903	-5.600505162
78	H	0.671139681	-1.993171399	-5.190926865
79	C	3.650600293	-0.507972490	-7.212839920
80	H	2.203784128	0.831717131	-8.076551784
81	C	3.855837973	-1.570233348	-6.324611198
82	H	2.944196185	-2.923562938	-4.897006056
83	H	4.486837571	-0.088474818	-7.775312991
84	C	-1.114372946	-0.860999208	-6.665867514
85	C	-1.181910181	-2.067133339	-7.379098723
86	C	-2.143220001	-0.507933000	-5.781478047
87	C	-2.268278617	-2.915986658	-7.215002229
88	H	-0.377986709	-2.335943740	-8.065870142
89	C	-3.230356455	-1.352289837	-5.619445992
90	H	-2.087740927	0.421557654	-5.214553781
91	C	-3.296226255	-2.561426959	-6.333364939
92	H	-2.318773920	-3.851715016	-7.774737565
93	H	-4.027852502	-1.084651142	-4.924788072
94	N	-5.152411242	-2.079501707	-1.342854831
95	H	-5.538844424	-1.806375222	-0.437372193
96	N	0.741705345	5.510369024	-1.341838543
97	H	1.178790240	5.713853907	-0.441556382
98	N	4.354321384	-3.457984855	-1.345601272
99	H	4.310211310	-3.934416160	-0.444226401
100	N	-5.522888801	-0.457668295	3.351603217
101	H	-5.851167949	-0.049440494	4.224324079
102	N	2.408676851	5.047040364	3.316665954
103	H	2.942207745	5.122689264	4.181144297
104	N	3.203531640	-4.526804958	3.365780291
105	H	3.026681968	-5.001222881	4.248412128
106	N	-0.764345426	5.514210080	-6.083957057
107	H	-0.422947362	5.828456034	-5.172978000
108	N	5.134807399	-2.125422323	-6.070596665
109	H	5.226180547	-2.603806954	-5.171282123
110	N	-4.412713941	-3.395256203	-6.074012289
111	H	-4.869219121	-3.236288374	-5.172741903
112	C	-6.437985423	-0.899235779	2.414266602
113	C	2.478085204	6.058992644	2.377251411
114	C	4.022124856	-5.117308261	2.421039372
115	C	-5.943982459	-2.710427915	-2.275902252
116	C	0.567118119	6.506895774	-2.274756314
117	C	5.293519577	-3.823879040	-2.282140391
118	C	-4.955199191	-4.279384275	-6.985304119
119	C	-1.225678126	6.428192863	-7.010342394
120	C	6.163721731	-2.162964011	-6.990994495
121	O	1.976113922	5.937536961	1.276509320
122	O	-6.090497612	-1.253258933	1.302940239
123	O	4.133111135	-4.653189276	1.301927016
124	O	0.181657180	6.243419347	-3.403359512
125	O	-5.531638137	-2.918297576	-3.406123487
126	O	5.271499532	-3.351425120	-3.408307952

127	O	-1.606975299	6.077907870	-8.113013348
128	O	-4.444724725	-4.461438415	-8.076397899
129	O	6.057144971	-1.640679495	-8.086248471
130	C	3.205182701	7.317489612	2.782852283
131	H	2.535464427	8.192411812	2.608852455
132	H	3.453752491	7.353809245	3.860755825
133	C	-7.889082550	-0.910766792	2.829195093
134	H	-8.327829786	-1.905982122	2.581291078
135	H	-8.035928645	-0.794985569	3.920365872
136	C	4.761493856	-6.365988206	2.838155689
137	H	5.827155060	-6.278304733	2.519567442
138	H	4.798818303	-6.506503808	3.934980525
139	C	0.840134956	7.946848643	-1.904077632
140	H	1.520572437	8.378162597	-2.679246549
141	H	-0.110349637	8.519373091	-2.022324504
142	C	-7.336549601	-3.166455392	-1.902928643
143	H	-8.043804492	-2.747902738	-2.659704223
144	H	-7.389112889	-4.270141873	-2.055038464
145	C	6.380469476	-4.811201600	-1.920047545
146	H	6.345432575	-5.643696317	-2.664235879
147	H	7.365295523	-4.319182415	-2.101025832
148	C	-1.250479234	7.896706812	-6.636166182
149	H	-0.717111459	8.455633911	-7.441534676
150	H	-2.304163042	8.250969936	-6.701133011
151	C	-6.217327302	-5.030645689	-6.611429817
152	H	-6.968297932	-4.843451382	-7.415308534
153	H	-6.000619191	-6.120844195	-6.680253944
154	C	7.449801942	-2.871830593	-6.617296889
155	H	7.674142846	-3.608406357	-7.424835992
156	H	8.279383755	-2.131208595	-6.676894011
157	C	4.474116196	7.449607870	1.922081740
158	H	5.126545913	6.549650721	2.044785255
159	H	4.197265720	7.456198951	0.835002229
160	C	-8.623016573	0.197046817	2.053207478
161	H	-8.158441079	1.192465699	2.262559646
162	H	-8.485291785	0.043115590	0.950581236
163	C	4.102155318	-7.571985899	2.146776861
164	H	3.027755228	-7.651644587	2.446870244
165	H	4.079028462	-7.407748046	1.037243868
166	C	1.426067372	8.188427975	-0.509246845
167	H	0.728660050	7.807129129	0.280468373
168	H	2.365985699	7.590515851	-0.375086295
169	C	-7.816208257	-2.809783272	-0.491883006
170	H	-7.133310956	-3.251477338	0.277900875
171	H	-7.754305650	-1.702126186	-0.327633453
172	C	6.334382835	-5.381037013	-0.498571463
173	H	6.383561570	-4.554401031	0.255655399
174	H	5.347592582	-5.881526203	-0.312038150
175	C	-0.656616771	8.255584141	-5.270754025
176	H	-1.256055121	7.779355314	-4.450576313
177	H	0.372450796	7.816184961	-5.164631428
178	C	-6.822203571	-4.698832727	-5.244129948
179	H	-6.104580031	-4.973043640	-4.426754940
180	H	-6.964963352	-3.588672187	-5.139238821
181	C	7.461874917	-3.569452530	-5.254082821
182	H	7.344047704	-2.815099072	-4.432164314
183	H	6.568731342	-4.244864004	-5.152957204
184	C	5.188482763	8.638705027	2.270350630
185	C	-10.012476878	0.209842202	2.389870982
186	C	4.800191873	-8.780586025	2.458470508
187	C	1.700208934	9.576604869	-0.296588028
188	C	-9.158158483	-3.259296766	-0.280763706
189	C	7.398112063	-6.312924406	-0.280481257
190	C	-0.591093699	9.670403100	-5.074462976
191	C	-8.075542235	-5.356853283	-5.043034229
192	C	8.654193832	-4.332662914	-5.057903967
193	C	5.798562042	9.646527754	2.570566570
194	C	-11.194847586	0.225613869	2.674002022
195	C	5.383468949	-9.816337759	2.714624444
196	C	1.950076681	10.750212942	-0.096826444
197	C	-10.300769036	-3.628813808	-0.090175828
198	C	8.288762474	-7.115800406	-0.075762042
199	C	-0.504480953	10.867356110	-4.877026070
200	C	-9.153936403	-5.883139927	-4.843703323
201	C	9.645376883	-5.011112832	-4.862880671
202	C	6.485091267	10.766054968	2.910559310
203	C	-12.512097527	0.249547275	2.994652794
204	C	6.026370457	-10.974451033	3.005531268
205	C	2.250767614	12.055816849	0.111520752
206	C	-11.582897021	-4.024260305	0.107927320
207	C	9.268828746	-8.029722741	0.132489720
208	C	-0.386711518	12.199334357	-4.653607468
209	C	-10.366366233	-6.447749161	-4.622582151
210	C	10.734154283	-5.787698852	-4.645652964
211	C	7.095030994	11.770585479	3.221855940
212	C	-13.692244495	0.263617342	3.286576749
213	C	6.606358271	-12.009418785	3.271530505
214	C	2.526154895	13.226953897	0.287952735
215	C	-12.734023371	-4.376969641	0.276461807
216	C	10.144935463	-8.855313660	0.303948452
217	C	-0.275450415	13.391457486	-4.443474484
218	C	-11.456225857	-6.946490351	-4.418890283
219	C	11.704848171	-6.491715454	-4.444174783
220	C	7.801110291	12.965591994	3.592321673
221	H	7.629662339	13.788109464	2.857013024
222	H	7.503069027	13.316782876	4.607452948
223	C	-15.086243897	0.267893834	3.632314454
224	H	-15.697043497	-0.295086034	2.885751268
225	H	-15.257521979	-0.167236826	4.644366252
226	C	7.294612665	-13.227832531	3.595050223
227	H	8.083057208	-13.472559346	2.843101174
228	H	7.762034026	-13.174324573	4.605939681
229	C	2.904691503	14.601822584	0.468286404
230	H	2.351124560	15.284909742	-0.217888537
231	H	2.745930103	14.945676206	1.513828838
232	C	-14.111325686	-4.750734949	0.450985466
233	H	-14.411853452	-5.579453168	-0.232207505
234	H	-14.330709005	-5.056826863	1.497512824
235	C	11.148791886	-9.870796801	0.471780317
236	H	12.005041501	-9.732556133	-0.229271410
237	H	11.542796689	-9.904201955	1.510832374
238	C	-0.122926452	14.799624735	-4.206136157
239	H	-0.961926550	15.391010015	-4.638750230
240	H	-0.047323971	15.029643950	-3.119081278
241	C	-12.753549789	-7.518633559	-4.190817932
242	H	-12.841228139	-8.542725358	-4.620570297
243	H	-13.000120124	-7.564958968	-3.105767291
244	C	12.839572488	-7.342213001	-4.219572102
245	H	13.769821811	-6.928417098	-4.671488385
246	H	13.016888541	-7.516972894	-3.134122268

247	O	-15.498892378	1.632662994	3.619918010
248	O	9.188175881	12.635136838	3.588421082
249	O	6.310420385	-14.259252412	3.570031995
250	O	-14.860497061	-3.594340043	0.081860325
251	O	4.284437806	14.654710616	0.109646931
252	O	10.500020627	-11.092397100	0.123105266
253	O	-13.673078015	-6.659930243	-4.865493584
254	O	1.086233552	15.168170034	-4.869536525
255	O	12.525043432	-8.574167891	-4.868841073
256	C	-16.857126919	1.824434178	4.019508223
257	H	-17.483379212	0.973646430	3.671845184
258	C	10.034485737	13.705772389	4.012864186
259	H	9.618506978	14.680981947	3.676989459
260	C	6.804507326	-15.534156415	3.983005881
261	H	7.855679106	-15.665924665	3.644501966
262	C	-16.194494351	-3.556175981	0.580799150
263	H	-16.655809886	-4.564844607	0.513726418
264	C	4.996048389	15.782548438	0.610851645
265	H	4.368229674	16.695961517	0.531020346
266	C	11.125784923	-12.271371858	0.620569326
267	H	12.229789369	-12.185377808	0.526018950
268	C	-15.043631853	-6.989972287	-4.650088370
269	H	-15.169887214	-8.093860398	-4.632779263
270	C	1.484962547	16.519066453	-4.646310751
271	H	0.592566906	17.180886416	-4.631315636
272	C	13.484313387	-9.606932902	-4.653289394
273	H	14.510370175	-9.180627519	-4.667992752
274	C	10.153939542	13.651352855	5.543912443
275	H	9.144672480	13.581760139	5.992814570
276	H	10.670693665	12.721753591	5.848497306
277	C	-16.896383024	1.953272479	5.550022541
278	H	-16.335872826	1.115605661	6.007587732
279	H	-16.358841279	2.867238168	5.865652967
280	C	6.699274930	-15.621158079	5.513228045
281	H	7.142996883	-14.715122580	5.968891534
282	H	5.636711649	-15.607900963	5.820518514
283	C	5.389707897	15.513438099	2.072012546
284	H	4.483561656	15.489856747	2.705969342
285	H	5.825890957	14.498474385	2.148657577
286	C	-16.165749485	-3.060756265	2.035540880
287	H	-15.669753988	-3.812861447	2.677309258
288	H	-15.528364075	-2.157264776	2.097546133
289	C	10.716136197	-12.470652316	2.088441708
290	H	11.178322847	-11.685820164	2.716124298
291	H	9.623767335	-12.315428918	2.183590522
292	C	2.256409884	16.591433420	-3.319119724
293	H	1.693818443	16.057348471	-2.529863335
294	H	3.213454342	16.041987185	-3.410682693
295	C	-15.501644836	-6.354669941	-3.327764363
296	H	-14.764187980	-6.573138778	-2.532137497
297	H	-15.503449798	-5.251350468	-3.422128201
298	C	13.181510469	-10.287026152	-3.309052621
299	H	13.052353113	-9.519736738	-2.522097685
300	H	12.206343502	-10.808934876	-3.364083199
301	C	11.354348065	13.399452340	3.307366895
302	H	11.229551882	13.407300780	2.217228530
303	H	12.126003927	14.130316268	3.569048769
304	H	11.727629949	12.403593151	3.578377459
305	C	-17.245122212	3.118611349	3.306924511
306	H	-17.166750482	3.007104139	2.218074465
307	H	-18.270876212	3.414443206	3.547886185
308	H	-16.580127670	3.943914714	3.591874134
309	C	5.871605899	-16.510663103	3.269221897
310	H	5.947648857	-16.402571225	2.179971266
311	H	6.106362087	-17.547832708	3.527750794
312	H	4.823149838	-16.325766711	3.536104899
313	C	6.198168926	15.853434007	-0.332367815
314	H	5.874599342	15.784946528	-1.380445607
315	H	6.749302543	16.790146071	-0.210447613
316	H	6.891182689	15.020276979	-0.160361159
317	C	-16.872152076	-2.573245481	-0.375155893
318	H	-16.648642236	-2.829981038	-1.420145345
319	H	-17.958739625	-2.578278487	-0.251741003
320	H	-16.511840356	-1.548843980	-0.217062284
321	C	10.573505993	-13.350798490	-0.311705115
322	H	10.666829013	-13.042239023	-1.362472733
323	H	11.107857463	-14.297406397	-0.190772256
324	H	9.506735113	-13.530734400	-0.127864356
325	C	2.370060723	16.815326720	-5.857252559
326	H	1.809042950	16.693749320	-6.792521333
327	H	2.762245973	17.836108762	-5.824837787
328	H	3.222395289	16.124701564	-5.904867255
329	C	-15.736431535	-6.375936909	-5.866765964
330	H	-15.339324661	-6.797346697	-6.798968912
331	H	-16.815448815	-6.555435234	-5.844012628
332	H	-15.573136521	-5.291134172	-5.912279026
333	C	13.256833859	-10.535472068	-5.846292264
334	H	13.408035424	-10.001816100	-6.793267592
335	H	13.940665090	-11.389196462	-5.823386835
336	H	12.229519292	-10.922575136	-5.858982283
337	C	10.892982958	14.876886475	6.088659399
338	H	11.954734477	14.853449958	5.778702806
339	H	10.470886199	15.804270795	5.654210936
340	C	-18.336817133	1.972330929	6.068157743
341	H	-18.846468396	2.901518104	5.750984452
342	H	-18.916588403	1.141186978	5.620840917
343	C	7.396486227	-16.876313757	6.043512401
344	H	6.849193343	-17.783090300	5.724208644
345	H	8.410875903	-16.964890868	5.606987378
346	C	6.371345069	16.568964226	2.586917806
347	H	7.348031324	16.458757205	2.076505496
348	H	6.012552928	17.583699718	2.329692038
349	C	-17.576436634	-2.768212370	2.552624066
350	H	-17.994449547	-1.883936324	2.033077684
351	H	-18.255657940	-3.607062155	2.308831033
352	C	11.115781455	-13.858625527	2.594665076
353	H	10.508078594	-14.635420327	2.090835473
354	H	12.165968577	-14.077709865	2.323192290
355	C	2.505545496	18.043306864	-2.902174882
356	H	3.105704395	18.564243127	-3.672386324
357	H	1.550547823	18.597216066	-2.838984896
358	C	-16.886976803	-6.863599511	-2.920705150
359	H	-17.632077573	-6.604798422	-3.697001492
360	H	-16.890313045	-7.967537496	-2.856474850
361	C	14.294894445	-11.262791499	-2.919314935
362	H	14.390734204	-12.057990259	-3.682985527
363	H	15.272437026	-10.746362796	-2.897845258
364	C	10.796101800	14.932441882	7.619436412
365	H	9.733568330	14.955879421	7.927597474
366	H	11.219364988	14.012836714	8.063818568

367	C	-18.363547962	1.856731404	7.598094542
368	H	-17.840696070	0.934018207	7.914074269
369	H	-17.803671120	2.692883644	8.056021193
370	C	7.495407696	-16.835903375	7.574048382
371	H	8.051797015	-15.933119610	7.890503756
372	H	6.488996719	-16.742104739	8.022001686
373	C	6.558739786	16.457643024	4.105184441
374	H	5.618117486	16.722185345	4.623489391
375	H	6.773175349	15.412917370	4.394101633
376	C	-17.572519735	-2.531203351	4.068299565
377	H	-17.294985526	-3.462630402	4.596412896
378	H	-16.799108192	-1.791370062	4.342771804
379	C	10.942983471	-13.964661700	4.114864189
380	H	11.654208777	-13.288820545	4.625574385
381	H	9.937027603	-13.620341280	4.415823261
382	C	3.231687835	18.090941297	-1.552195030
383	H	2.606551252	17.617010813	-0.772288516
384	H	4.162766941	17.492836399	-1.608982034
385	C	-17.301822036	-6.257097728	-1.574574022
386	H	-16.585756762	-6.561495289	-0.788331371
387	H	-17.247995267	-5.151813944	-1.631732047
388	C	13.997794908	-11.885664217	-1.549985651
389	H	13.953786028	-11.094194693	-0.778367447
390	H	12.995845313	-12.358846283	-1.567182977
391	C	11.527552284	16.166252827	8.163378689
392	H	11.250140661	17.061263537	7.574379103
393	H	12.619378566	16.050074008	8.035167527
394	C	-19.808362422	1.844238624	8.113217292
395	H	-20.418219002	1.142022794	7.513369279
396	H	-20.272268437	2.837808485	7.973515020
397	C	8.192649867	-18.096570796	8.101180766
398	H	9.093344352	-18.313230607	7.495667674
399	H	7.533109659	-18.975466177	7.980894838
400	C	7.695440003	17.374653882	4.573468896
401	H	7.485012551	18.419450704	4.278193496
402	H	8.639904004	17.104076801	4.064591008
403	C	-18.949545949	-2.047320994	4.539711741
404	H	-19.727942322	-2.775938551	4.245970184
405	H	-19.216946740	-1.101664566	4.031424130
406	C	11.162743981	-15.409982979	4.578373646
407	H	12.165032181	-15.759212054	4.267666352
408	H	10.442448009	-16.085766386	4.079765036
409	C	3.561577821	19.533559679	-1.153478916
410	H	2.633986925	20.131181823	-1.083003927
411	H	4.169449624	20.016730616	-1.941282613
412	C	-18.719992953	-6.690734337	-1.187598539
413	H	-19.435557750	-6.404033480	-1.980900159
414	H	-18.776368486	-7.792806933	-1.118520889
415	C	15.058765538	-12.925452102	-1.172958539
416	H	16.060582300	-12.458109724	-1.152279965
417	H	15.112210149	-13.713255974	-1.947493196
418	C	11.191607790	16.401751207	9.642090658
419	H	11.560579788	15.559387729	10.256324715
420	H	10.093943614	16.419623297	9.783304558
421	C	-19.862395915	1.438932392	9.592143091
422	H	-19.304385426	0.495702631	9.747298702
423	H	-19.350609112	2.197667106	10.213107260
424	C	8.589750955	-17.929896148	9.573743192
425	H	7.685746751	-17.805471736	10.198629100
426	H	9.177140619	-17.000769581	9.704057914
427	C	7.880308017	17.286711302	6.093803461
428	H	8.197186947	16.264524265	6.379334186
429	H	6.915289293	17.457101744	6.606683105
430	C	-18.969765178	-1.844542303	6.060249531
431	H	-18.617983734	-2.759502585	6.572504587
432	H	-18.257906216	-1.045925245	6.346993116
433	C	11.016155764	-15.525363916	6.100963893
434	H	9.978721408	-15.280709633	6.401972650
435	H	11.659741066	-14.779972314	6.604516378
436	C	4.313671067	19.572577192	0.183903261
437	H	3.704878395	19.097127767	0.976066356
438	H	5.243365904	18.975814354	0.113209554
439	C	-19.139411806	-6.059293662	0.147008311
440	H	-19.081721708	-4.955901221	0.078732464
441	H	-18.431877054	-6.353282980	0.945241899
442	C	14.744566158	-13.554384641	0.191455407
443	H	14.695330407	-12.768814442	0.969168008
444	H	13.743319187	-14.025953114	0.169300339
445	C	11.796836445	17.718496636	10.128911359
446	H	11.399809362	18.571201553	9.566119016
447	H	11.576254437	17.892657142	11.187765586
448	H	12.886441179	17.727918029	10.013578780
449	C	-21.309218818	1.265487674	10.053966313
450	H	-21.817685645	0.477315193	9.486882550
451	H	-21.359499018	0.991541772	11.113616787
452	H	-21.886547983	2.187621529	9.924270444
453	C	9.405185165	-19.129465080	10.055862448
454	H	10.329001036	-19.243710173	9.477196476
455	H	9.689007821	-19.021257205	11.108576086
456	H	8.840947355	-20.063860450	9.961277893
457	C	8.912202841	18.306923925	6.573807837
458	H	8.580700828	19.334010636	6.386101764
459	H	9.094390397	18.211054694	7.650801480
460	H	9.874265770	18.174341135	6.065258717
461	C	-20.376235206	-1.487427977	6.540188808
462	H	-21.086198957	-2.298971611	6.347219157
463	H	-20.389266615	-1.287947692	7.618339147
464	H	-20.756806810	-0.591478854	6.036050511
465	C	11.379935466	-16.931997561	6.575166189
466	H	12.430205429	-17.167053501	6.371059078
467	H	11.221382724	-17.040584693	7.654704094
468	H	10.769940465	-17.693929463	6.076064514
469	C	4.648632476	21.011007673	0.576950343
470	H	3.743204103	21.619028668	0.683999129
471	H	5.185574627	21.050650665	1.531139327
472	H	5.280298465	21.496048507	-0.176141059
473	C	-20.558129532	-6.482503323	0.526340488
474	H	-20.638671103	-7.570562119	0.629206195
475	H	-20.866874051	-6.038884639	1.479362155
476	H	-21.286008017	-6.171774447	-0.231922535
477	C	15.799113915	-14.594474689	0.567679516
478	H	16.799324481	-14.150251023	0.624048604
479	H	15.585247282	-15.046243326	1.542689665
480	H	15.843157646	-15.405686463	-0.167966264
